# A Self‐Separating Multiphasic System for Catalytic Hydrogenation of CO_2_ and CO_2_‐Derivatives to Methanol

**DOI:** 10.1002/cssc.202201250

**Published:** 2022-10-26

**Authors:** Thomas Diehl, Patrick Lanzerath, Giancarlo Franciò, Walter Leitner

**Affiliations:** ^1^ RWTH Aachen University Institut für Technische und Makromolekulare Chemie (ITMC) Worringerweg 2 52074 Aachen Germany; ^2^ Max-Planck-Institut für chemische Energiekonversion Stiftstraße 34–36 45470 Mülheim a. d. Ruhr Germany

**Keywords:** biphasic catalysis, CO_2_ hydrogenation, homogeneous catalysis, ligand design, methanol

## Abstract

Catalytic conversion of CO_2_ and hydrogen to methanol was achieved in a self‐separating multiphasic system comprising the tailor‐made complex [Ru(CO)ClH(MACHO‐C_12_)] (MACHO‐C_12_=bis{2‐[bis(4‐dodecylphenyl)phosphino]ethyl}amine) in *n*‐decane as the catalyst phase. Effective catalyst recycling was demonstrated for the carbonate and the amine‐assisted pathway from CO_2_ to methanol. The polar products MeOH or MeOH/H_2_O generated from the catalytic reactions spontaneously formed a separate phase, allowing product isolation and catalyst separation without the need for any additional solvent. In the amine‐assisted hydrogenation of CO_2_, the catalyst phase was recycled over ten subsequent runs, reaching a total turnover number to MeOH of 19200 with an average selectivity of 96 %.

## Graphical abstract

**Phase separation**: Ru‐catalyzed hydrogenation of dimethylcarbonate or CO_2_ to methanol was accomplished in *n*‐decane using the new alkyl‐tagged MACHO‐C_12_ nonpolar ligand. During the reaction, the polar products MeOH or MeOH/H_2_O naturally separate in a second phase. In the amine‐assisted hydrogenation of CO_2_, the catalyst phase was recycled for 10 times, reaching a total turnover number to MeOH of 19200.

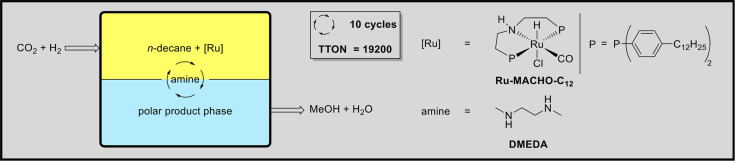

## Introduction

**Scheme 1:**
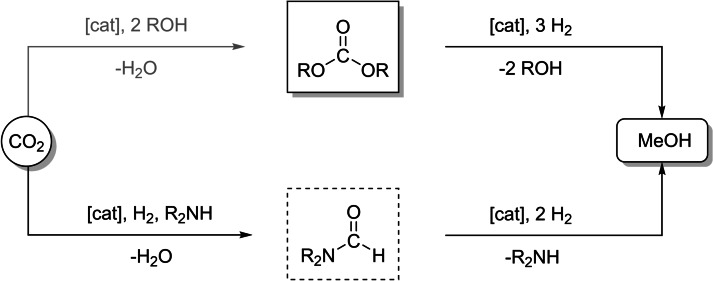
Indirect pathways for CO_2_ hydrogenation to methanol using organic carbonates as intermediates (top) or amine‐assisted via in situ formed formamides (bottom) investigated in this study.

The use of organometallic complexes for hydrogenation of carbon dioxide (CO_2_) is a longstanding research field of great current interest.[^1^, ^2^] Activities in this field are stimulated by the scientific challenge to selectively hydrogenate the notoriously unreactive substrate CO_2_ as well as by potential ramifications for innovative processes at the energy‐chemistry nexus.^[3]^ Among the possible hydrogenation products accessible from CO_2_, methanol is finding particular interest due to its many possible uses as base chemical, intermediate, or energy carrier.^[4]^ Significant progress has been made recently to identify lead structures of organometallic catalysts for the conversion of CO_2_ and hydrogen to methanol either directly or via indirect pathways using isolated or in situ generated organic intermediates.[^5^, ^6^] The use of organic carbonates[^6a^, ^7^] and the amine‐assisted formation of formamides^[8]^ are prominent examples for the indirect pathways (Scheme 1). While many studies deal with the search for new catalyst structures and their mechanistic understanding, comparably few efforts are devoted to the down‐stream integration of effective product isolation and catalyst recycling.[^6e^, ^8b^, ^8c^, ^8e^] These two aspects are critical for the implementation of such processes on any scale, however, as they define the resource and energy efficiency of the overall process.

**Figure 1:**
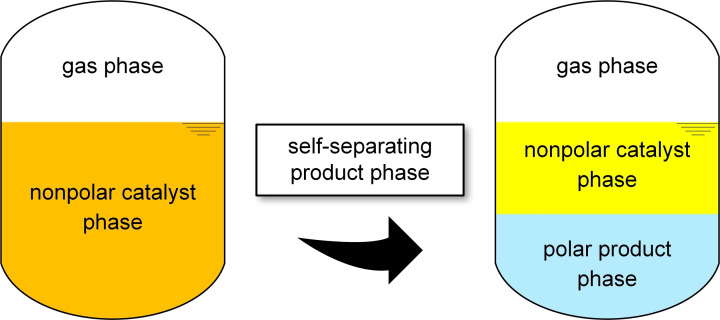
Envisaged multiphasic catalytic system with self‐separating product phase for the reactions shown in Scheme 1.

Highly polar product phases consisting of methanol or mixtures of methanol and water are formed inherently during catalytic CO_2_ hydrogenation. We thus envisaged the possibility to conduct the catalytic transformation in a nonpolar organic solvent from which the product phase would self‐separate spontaneously (Figure 1). In this paper, we demonstrate this concept for the Ru‐catalyzed hydrogenation of CO_2_ via organic carbonates exemplified by dimethyl carbonate (DMC) and via the amine‐assisted pathway using *N*,*N*′‐dimethylethane‐1,2‐diamine (DMEDA).

## Results and Discussion

**Scheme 2:**
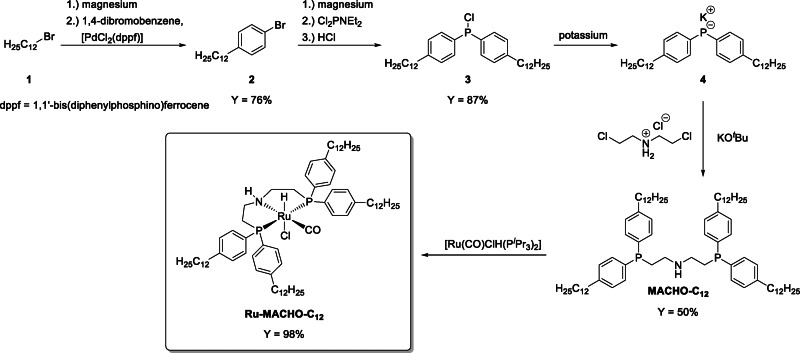
Reaction pathway for the synthesis of the tagged ligand MACHO‐C_12_ and the corresponding Ru‐MACHO‐C_12_ pre‐catalyst.

Based on our previous experience with CO_2_ hydrogenation to formates,^[9]^ *n*‐decane was chosen as catalyst phase. Given its versatility in CO_2_ hydrogenation to methanol and related reactions, the complex [Ru(CO)ClH(HN(CH_2_CH_2_PPh_2_)_2_)] (Ru‐MACHO) was selected as lead structure.[^8a^, ^8b^, ^8c^, ^8d^] The MACHO ligand was tagged with long alkyl chains to ensure its exclusive partitioning into the nonpolar phase under biphasic conditions. The synthesis of the modified MACHO‐C_12_ ligand and the corresponding complex [Ru(CO)ClH(MACHO‐C_12_)] (Ru‐MACHO‐C_12_) used as pre‐catalyst is shown in Scheme 2. DMEDA was chosen because it is one of the best‐performing amines for the assisted hydrogenation of CO_2_ to MeOH as reported in the literature.^[8d]^ The detailed procedures are summarized in the Experimental Section.

Starting from the commercially available 1‐bromododecane (**1**) and 1,4‐dibromobenzene, the compound 1‐bromo‐4‐dodecylbenzene (**2**) was obtained in good yields (76 %) by Kumada coupling.^[10]^ The Grignard reagent of **2** was reacted with dichloro(diethylamino)phosphine followed by the addition of hydrochloric acid to give chloro‐bis(4‐dodecylphenyl)phosphine (**3**) in high yields (87 %).^[11]^ Treating **3** with elemental potassium gave **4**, which was reacted further without isolation. A solution of bis(2‐chloroethyl)amine hydrochloride, deprotonated in situ with KO^*t*^Bu, was added resulting in the desired nonpolar ligand bis{2‐[bis(4‐dodecylphenyl)phosphino]ethyl}amine (MACHO‐C_12_) in moderate yields (50 %).^[12]^ Finally, complexation of the ligand by phosphine exchange of the known complex [Ru(CO)ClH(P^*i*^Pr_3_)_2_] led to the tagged pre‐catalyst [Ru(CO)ClH(MACHO‐C_12_)] (Ru‐MACHO‐C_12_) isolated as dark orange viscous oil in quantitative yield as mixture of *syn* and *anti* isomers.^[13]^

The spectroscopic data of the ligand and the new complex Ru‐MACHO‐C_12_ are fully in line with the data of the unmodified parent compounds, reflecting the slight electron donating effect of the alkyl substituent in *para*‐position of the aromatic rings. In the ^31^P nuclear magnetic resonance (NMR) spectra, the signal of the tagged MACHO‐C_12_ at −23.0 ppm shows an upfield shift as compared to the unmodified MACHO at −20.7 ppm.^[14]^ Also, the comparison of the ^31^P NMR signals of the *syn* and *anti* isomers of the corresponding Ru‐MACHO‐C_12_ and Ru‐MACHO complexes (50.9 and 53.4 ppm vs. 53.7 and 57.0 ppm) show a consistent upfield shift, while no major differences are displayed by the characteristic triplets in the hydride region in the ^1^H NMR spectra (−14.76 and −13.88 ppm vs. −15.01 and −14.30 ppm).^[13]^

**Figure 2:**
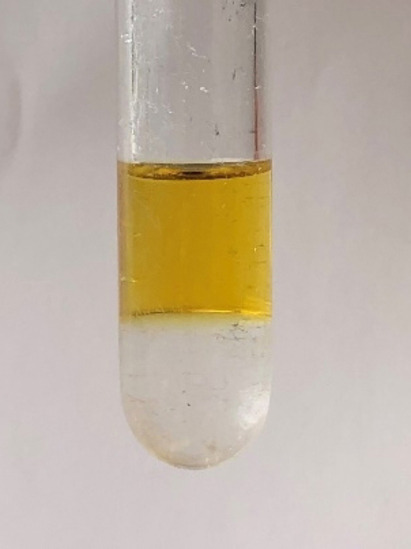
Observed partitioning of the Ru‐MACHO‐C_12_ complex (53.90 mg) in a liquid–liquid biphasic mixture comprising *n*‐decane (top, 2 mL) and an equimolar mixture of methanol and water (bottom, 1.42 mL/0.63 mL).

While the untagged Ru‐MACHO complex is almost insoluble in *n*‐decane, the Ru‐MACHO‐C_12_ complex was found to be highly soluble in *n*‐decane. A biphasic mixture was obtained when an equimolar mixture of MeOH and water was added to a *n*‐decane solution of Ru‐MACHO‐C_12_ with the color of the complex retained exclusively in the organic phase (Figure 2).

Analysis of the aqueous phase by inductively coupled plasma mass spectrometry (ICP‐MS) revealed a content of ruthenium of 1.9 ppm and phosphorus of 8.7 ppm, corresponding to less than 0.05 and 0.25 %, respectively, of the charged metal complex. The preferential partition of the Ru‐complex in the organic phase was also reflected by the NMR analysis of the aqueous phase where no signals in ^31^P and ^1^H NMR spectra could be detected (Figures S15‐17). The NMR spectra of the organic phase exhibited only the unperturbed signals of the pre‐catalyst (Figures S12‐14). These data confirm the desired partitioning and the necessary robustness of the pre‐catalyst in the presence of the expected polar product mixtures.

**Scheme 3:**
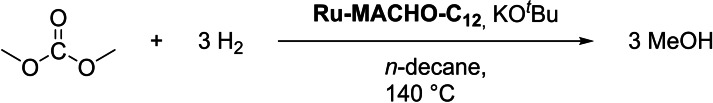
Liquid–liquid multiphasic hydrogenation of DMC to methanol using the tailored Ru‐MACHO‐C_12_ pre‐catalyst.

**Figure 3:**
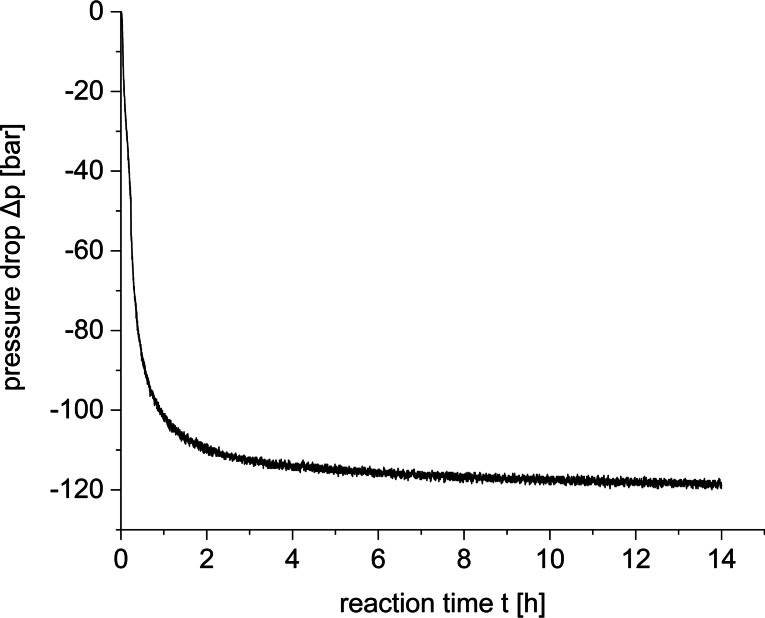
Pressure/time profile for the liquid–liquid multiphasic hydrogenation of DMC to methanol using the tailored Ru‐MACHO‐C_12_ pre‐catalyst. Reaction conditions: *n*(Ru)=30 μmol, *n*(DMC)=30 mmol, *n*(KO^*t*^Bu)=30 μmol, *V*(*n*‐decane)=2.5 mL, *p*(H_2_)=120 bar, *T=*140 °C, *t=*14 h.

**Figure 4:**
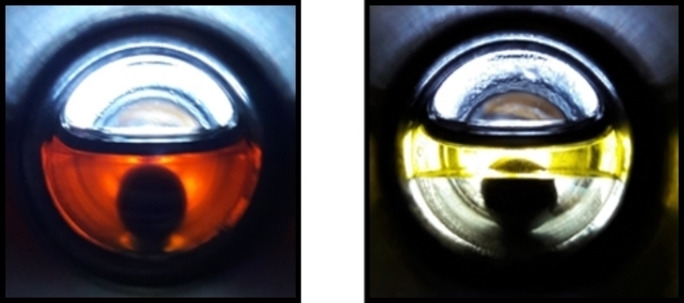
Observed reaction solution before (left) and after a typical hydrogenation experiment using dimethyl carbonate as substrate, where the spontaneously formed liquid product phase is clearly visible (right).

The concept of integrated product separation was tested first for the hydrogenation of dimethyl carbonate (DMC) to methanol (Scheme 3). The catalytic experiments were carried out in a 10 mL high pressure reactor equipped with thick‐walled borosilicate windows for optical monitoring of the phase behavior. For the catalytic reactions, the Ru‐MACHO‐C_12_ (30 μmol) was dissolved in *n*‐decane (2.5 mL) and activated with KO^*t*^Bu (30 μmol) to form the active Ru‐amide complex.^[15]^ A clear dark red solution was obtained after addition of DMC (2.7 g, 30 mmol) (Figure 4, left). The reactor was pressurized with H_2_ to 120 bar at room temperature and heated to 140 °C. The progress of the reaction was monitored online through the pressure changes in the reactor (Figure 3).

The recorded pressure curve shows a rapid pressure drop right from the beginning. After approximately 4 h, almost no further pressure decrease was observed corresponding to a total pressure loss of approximately 118 bar after 14 h, indicating practically complete consumption of the initially charged H_2_ (120 bar) at that stage. The reaction was stopped by cooling the reactor to room temperature after no further pressure change was detected. The reaction mixture showed two clearly separated liquid phases with the yellow color indicative of the metal complex preferentially in the upper phase (Figure 4, right). Under these reaction conditions, a conversion of 50 % was obtained for DMC corresponding to a turnover number (TON) of 404 based on the reduction of the carbon atom of the carbonyl group to methanol. Of the formed methanol, 85 % was found in the product phase and correspondingly 15 % remained in the catalyst phase. Similarly, the remaining DMC partitioned between the two phases in a ratio of 77 to 23 %. Therefore, the reaction was repeated with a lower substrate loading (10 mmol) and 200 bar hydrogen pressure (RT) under otherwise identical reaction conditions reaching a conversion of >99 % after 24 h reaction time.

**Scheme 4:**
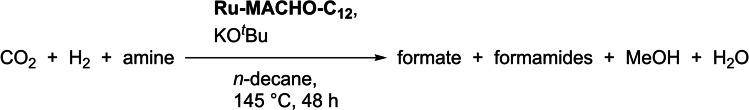
Liquid–liquid multiphasic hydrogenation of CO_2_ to methanol via the amine‐assisted pathway using the tailored Ru‐MACHO‐C_12_ pre‐catalyst.

**Figure 5:**
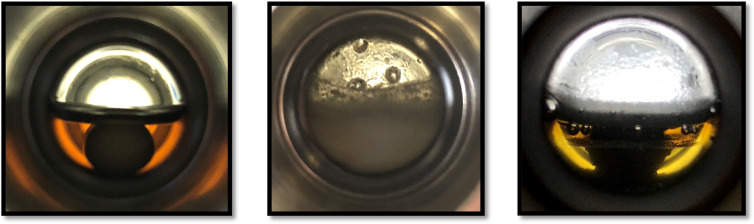
Observed reaction solution for amine‐assisted CO_2_ hydrogenation before reaction (left), after saturation with carbon dioxide (middle), and after a typical hydrogenation experiment (right).

**Figure 6:**
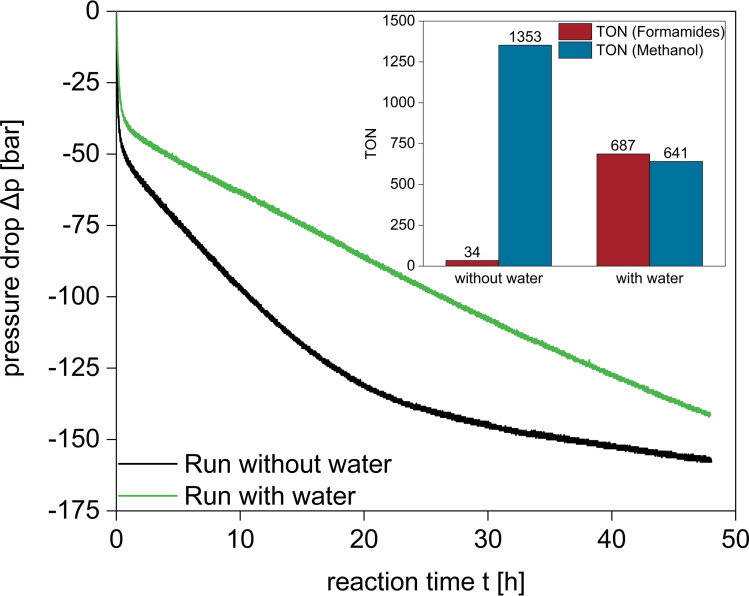
Pressure/time profiles for the liquid–liquid multiphasic hydrogenation of CO_2_ to methanol via the amine‐assisted pathway using the tailored Ru‐MACHO‐C_12_ pre‐catalyst with and without additional water. Reaction conditions: *n*(Ru)=5 μmol, *n*(DMEDA)=10 mmol, *n*(KO^*t*^Bu)=0.01 mmol, *V*(*n*‐decane)=2.5 mL, *V*(H_2_O)=0.4 mL (if present), *p*(CO_2_)=20 bar for 10 min while stirring for saturation, then vented, *p*(H_2_)=200 bar, *T=*145 °C, *t=*48 h.

In order to evaluate the same approach for the amine‐assisted pathway for CO_2_ hydrogenation (Scheme 4), the catalyst solutions were prepared in the same way as described above. As amine component, DMEDA (10 mmol) was added forming a clear solution with the catalyst phase (Figure 5, left). After saturation of the amine with CO_2_ (20 bar, RT, 10 min), the resulting carbamate salts precipitated from the nonpolar solvent (Figure 5, middle). The CO_2_ pressure was released and exchanged for hydrogen (200 bar, RT) followed by heating the reaction mixture to 145 °C and switching on the stirrer to start the reaction. The carbamate salt was melting and/or partly reconverting to the amine at the reaction temperature leading to a liquid–liquid biphasic system from the beginning. The progress of the reaction was monitored online by the pressure change in the reactor (Figure 6), and a two‐phase mixture was again observed once the curve had flattened out.

The phases were readily separated (Figure 5, right) and the composition of the reaction mixture analyzed by NMR spectroscopy with internal standards. The analysis of the product phase confirmed a high methanol content (6.8 mmol) with only small amounts of formamides (<2.5 %) and traces of formates present after 48 h reaction time. DMEDA partitioned between the product phase (63 %) and the catalyst phase (37 %).

The total amount of methanol corresponds to a TON of 1353 for CO_2_ conversion to MeOH. After a very fast initial pressure drop within the first hour, the rate of hydrogen consumption was nearly linear indicating a turnover frequency (TOF) of 42 h^−1^ before hydrogen consumption leveled off after around 25 h. The two regimes can be assigned to the fast hydrogenation to the formate level and the subsequent slow hydrogenation via formamide to methanol, consuming one and two equivalents of H_2_ per CO_2_, respectively, in line with the relative pressure drops of around 50 and 100 bar (Figure 6).

Next, we compared the self‐separating system to a biphasic *n*‐decane/water system where water is added as additional solvent from the beginning to act as the product phase. Interestingly, the second hydrogenation step was significantly faster in the absence of the water phase (Figure 6, black vs. green line). When water was present from the beginning, a TOF of only 23 h^−1^ was observed for the reduction beyond the formate level. While the overall TON for CO_2_ conversion was with 1328 close to the self‐separating system, a much lower selectivity of only around 50 % for the full hydrogenation to methanol was observed leaving large amounts of formamide present at the end of the reaction.

**Figure 7:**
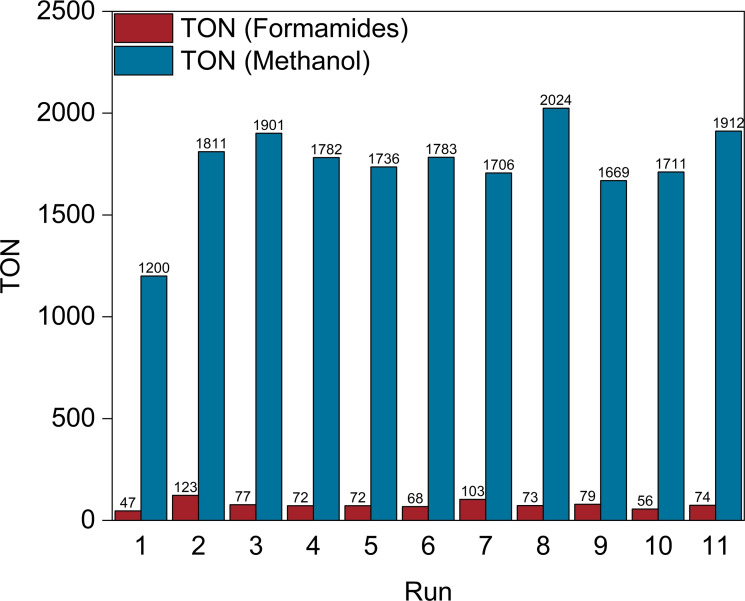
Recycling experiments for the amine‐assisted CO_2_ hydrogenation in the self‐separating system. Reaction conditions: *n*(Ru)=5 μmol, *n*(DMEDA)=10 mmol (added prior to each run), *n*(KO^*t*^Bu)=0.01 mmol, *V*(*n*‐decane)=2.5 mL, *p*(CO_2_)=20 bar for 10 min while stirring for saturation, then vented, *p*(H_2_)=200 bar, *T=*145 °C, *t=*48 h. TONs were determined by ^1^H NMR spectroscopy using 1,4‐dioxane (for product phase) and mesitylene (for catalyst phase) as internal standard and are given relative to the initial catalyst loading.

As the self‐separating system led to a high single‐pass TON with high selectivity to methanol and effective product separation, it was chosen to investigate the robustness of the catalyst system by repetitive batch recycling of the catalyst phase (Figure 7).

After 48 h reaction time, the reactor was cooled to room temperature and the pressure released. Under argon atmosphere, the product phase was carefully withdrawn by syringe leaving the catalyst phase behind. The product phase was analyzed by NMR spectroscopy and ICP‐MS to determine its composition and potential catalyst leaching. The reactor was loaded again with DMEDA, and the procedure was repeated. The amount of methanol isolated from the first run corresponded to a TON of 1200 with only small amounts of formamide. As observed in the batch reactions, small amounts of methanol, formamide, and DMEDA remained in the *n*‐decane phase saturating it with these components for the subsequent runs. Consequently, the amounts of methanol isolated in the following runs correspond to a higher TON relative to the initially charged catalyst loading averaging around 1800 in all further recycling experiments. In total, the procedure was repeated over eleven subsequent runs reaching a total TON (TTON) of 19235 mol methanol per mol ruthenium charged initially at a constant high average selectivity of 96 % over the formamide intermediate.

The ICP‐MS data indicated a significant leaching of ruthenium into the aqueous phase in the first two cycles corresponding to around 20 % of the charged catalyst (Table S1). Leaching was much lower in the following nine cycles with values between 0.6 and 3.6 %. Over the eleven cycles, a total of 37 % of the ruthenium was depleted, but the system still reached very similar amounts of product throughout the entire series of experiments. Repeating the series with a new catalyst batch showed largely identical behavior leading to a TTON of 15859 albeit at a slighter reduced methanol selectivity (85 %) mainly due to a somewhat higher amount of remaining formamide product in the initial runs (Table S2). The robustness of the method was validated by recycling experiments in a different reactor set‐up (10 mL, standard high pressure reactor without windows). At somewhat shorter reaction time (36 h), a TON_average_ of 1372 with an average selectivity of 91 % over four cycles confirmed the effective recycling.

Comparison with the state‐of‐the‐art in the literature shows that the presented system is among the best‐performing recycling concepts for homogeneously catalyzed hydrogenation of CO_2_ to methanol.^[16]^ While some catalytic systems lead to higher TONs in single batches, the intrinsic separation of the present system allows for very high total turnover numbers and hence effective use of the metal and ligand. For example, Everett and Wass^[17]^ reported a TON of up to 8900. Zhou and co‐workers^[18]^ achieved a TON of 9800 in the hydrogenation of *N*‐formylmorpholine, but only 2100 in the direct CO_2_ hydrogenation to methanol. A TON of 9900 for amine‐assisted CO_2_ hydrogenation to methanol was obtained by Prakash and co‐workers, also using the Ru‐MACHO framework.^[8d]^ The TTON of 19235 achieved in our present work also compares very favorably to previous attempts for recycling of methanol catalysts as presented by Leitner, Klankermayer and co‐workers (TTON: 796)^[6e]^ and Prakash and co‐workers (TTON: 2150^[8b]^ and 582^[8c]^).

## Conclusion

We demonstrated the possibility to exploit the inherent formation of very polar product mixtures during hydrogenation of CO_2_ or its derivatives to methanol for self‐separation into multiphasic catalytic systems. The concept relies on the tagging of a suitable catalyst lead structure with long alkyl chains for de‐facto immobilization in a nonpolar solvent such as *n*‐decane. The synthetic route to such ligands is readily accomplished by incorporating the tagged aryl phosphine building block in full analogy to the synthesis of the unmodified ligand. The widely used MACHO‐ligand and its Ru‐complex were used here to demonstrate this strategy using the hydrogenation of organic carbonates and the amine‐assisted CO_2_ hydrogenation pathway as proof‐of‐principle. The obtained productivity largely surpasses that of previous reports for homogeneously catalyzed CO_2_ hydrogenation to methanol[^6e^, ^8b^, ^8c^, ^8d^, ^16^, ^17^, ^18^] underlining the potential of the approach to exploit inherent product separation in multiphasic catalyst systems.^[19]^ Extension to other processes involving CO_2_ hydrogenation and implementation of (semi‐)continuous‐flow operation is currently investigated in our laboratories.

## Experimental Section

### General methods and chemicals

If not otherwise described, all reactions were carried out under exclusion of air and moisture using Schlenk techniques or by manipulation in a glovebox (Labmaster SP from the company MBraun) under argon (4.8, by Air Products) atmosphere. Used glassware was dried by heating it with a heat gun (630 °C) under vacuum (1×10^−3^ mbar) and cycling with argon for at least three times. Chemicals were purchased from abcr, Acros Organics, Alfa‐Aesar, Sigma Aldrich and TCI Chemicals. Dichloro‐(diethylamino)phosphine was used after distillation.^[11c]^ All other chemicals were used as received. 1‐Bromo‐4‐dodecylbenzene^[10]^ and [Ru(CO)ClH(P^*i*^Pr_3_)_2_]^[20]^ were synthesized according to literature procedures. Solvents for air and moisture sensitive reactions were deoxygenated by bubbling argon for at least 30 min and stored in Schlenk bottles over molecular sieves (3 or 4 Å). Hydrogen (5.0 by Air Products) and carbon dioxide (4.5 by Westfalen AG) were used as received.

Safety advice: High pressure experiments with compressed gases involve a significant risk and must be performed according to safety procedures and in conjunction with the use of suitable equipment.

### Analytical methods

^1^H, ^13^C, and ^31^P NMR spectra were recorded at room temperature using a Bruker Avance II 300 MHz, Bruker Avance II 400 MHz, or a Bruker Ascend 400 MHz spectrometer. Chemical shifts (*δ* in ppm) for ^1^H and ^13^C spectra are given relative to trimethylsilane and are referenced to the respective residual solvent signal,^[21]^ while ^31^P spectra are given relative to H_3_PO_4_ as external standard. Spin multiplicities are abbreviated as s (singlet), d (doublet), t (triplet), q (quartet), m (multiplet), br (broad). ICP‐MS measurements were performed by using water as matrix on an Agilent ICP‐MS triple quadrupole (Modell 8800). Samples were diluted to ensure a Ru concentration within the calibration (external standard). The lower detection limit was 1 ppb. CHN elemental analysis was carried out on an UNICUBE device by Elementar Analysensysteme GmbH. High resolution mass spectroscopy (HRMS) measurements were carried out on Thermo Fisher Scientific spectrometers Exactive™ Q Exactive Plus Orbitrap or on an LTQ FT Ultra, 7T FT‐ICR MS. Attenuated total reflectance infrared (ATR‐IR) spectra of the oily substances were recorded under argon atmosphere using a Bruker ALPHA FTIR.

### Synthesis of chloro‐bis(4‐dodecylphenyl)phosphine

The synthesis was carried out adapting literature‐known procedures.^[11]^ In a 500 mL two‐necked Schlenk flask with reflux condenser, magnesium turnings (3.14 g, 129.09 mmol, 2.0 equiv.) were suspended in tetrahydrofuran (100 mL). In another Schlenk tube, 1‐bromo‐4‐dodecylbenzene (21.00 g, 64.58 mmol, 1.0 equiv.) was dissolved in tetrahydrofuran (15 mL) and added to the suspension. After adding small quantities of iodine as magnesium activator, the reaction mixture was stirred for 3 h under reflux conditions and subsequently for 16 h at 60 °C. The Grignard solution was filtered through a frit filled with celite® to remove excess of magnesium turnings and the yield of the Grignard reagent was determined via ^1^H NMR spectroscopy. In a 500 mL two‐necked Schlenk flask with metering funnel, dichloro‐(diethylamino)phosphine (4.942 g, 28.10 mmol, 0.50 equiv.) was dissolved in THF (30 mL). The Grignard solution was transferred into the metering funnel via cannula and added dropwise to the phosphine solution at 0 °C. After the addition was complete, the reaction mixture was stirred for further 2 h at 0 °C. The reaction solution was warmed to room temperature and the solvent was removed under reduced pressure (1×10^−3^ mbar). To the residue, *n*‐pentane (100 mL) was added and after filtration through a frit filled with celite® a clear, colorless solution was obtained. The solution was cooled down to 0 °C followed by dropwise addition via syringe pump of a hydrochloride acid solution in diethyl ether (30.66 mL, 2 m, 61.32 mmol, 1.1 equiv.). Formed salts were filtered off via a cannula filter and a clear, colorless solution was obtained. The solvent was removed under reduced pressure (1×10^−3^ mbar) and the desired product was obtained as pale yellow, viscous oil, which crystallized overnight into a colorless powder (15.6 g, 27.99 mmol, 87 %). ^1^H NMR (400 MHz, chloroform‐*d*, 298 K): *δ*=0.89 (t, ^3^ *J*_H,H_=6.8 Hz, 6H, −CH_2_−C*H*_3_), 1.26 (br. m, 36H, −CH_2_−C_9_ *H*_18_−CH_3_), 1.61 (m, 4H, −CH_2_−C*H*_2_−C_10_H_21_), 2.62 (t, ^3^ *J*_H,H_=7.6 Hz, 4H, Ar−C*H*_2_−C_11_H_23_), 7.22 (d, ^3^ *J*_H,H_=6.8 Hz, 4H, Ar−*H*), 7.50 ppm (dd, ^3^ *J*_H,H_=6.8 Hz, ^3^ *J*_P,H_=7.6 Hz, 4H, Ar−*H*). ^13^C NMR (101 MHz, chloroform‐*d*, 298 K): *δ*=14.3 (s, −CH_2_−*C*H_3_), 22.9 (s, −*C*H_2_−), 29.5–29.9 (m, −*C*H_2_−), 31.4 (s, −CH_2_−*C*H_2_−C_10_H_21_), 32.1 (s, −*C*H_2_−), 36.0 (s, Ar−*C*H_2_−C_11_H_23_), 128.9 (d, ^3^ *J*_C,P_=7.0 Hz, Ar−*C*), 132.0 (d, ^2^ *J*_C,P_=24.7 Hz, Ar−*C*), 135.9 (d, ^1^ *J*_C,P_=31.9 Hz, P−*C*_q_), 145.8 ppm (s, Ar−*C*_q_). ^31^P{^1^H} NMR (162 MHz, chloroform‐*d*, 298 K): *δ*=83.4 ppm (s, Ar_2_−*P*−Cl). HRMS/ESI (pos): [C_36_H_59_ClP]^+^: calculated: 557.40374 *m*/*z*. found: 557.40381 *m*/*z*.

### Synthesis of potassium bis(4‐dodecylphenyl)phosphide

The synthesis was carried out adapting a literature procedure.^[12a]^ Elemental potassium (2.842 g, 72.69 mmol, 5.0 equiv.) was weighed into a 50 mL centrifuge Schlenk tube. In another Schlenk tube, a solution of chloro‐bis(4‐dodecylphenyl)phosphine in 2‐methyltetrahydrofuran (30 mL) was prepared and added to the elemental potassium. The reaction suspension was stirred vigorously at 75 °C. At this temperature, the potassium melted, and golden colored potassium spheres formed. After 2 h, the reaction solution turned deep red. The reaction progress was monitored via ^31^P NMR spectroscopy and after complete reaction, *n*‐pentane (15 mL) was added to the phosphide solution whereby excess of elemental potassium sinks to the bottom of the Schlenk tube. After centrifugation, the supernatant was decanted via syringe into another Schlenk tube, and the exact volume (33 mL) was noted. Next, triphenyl phosphine (21.3 mg, 0.081 mmol) as internal standard was weighed into an NMR tube and an exact volume (0.5 mL) of phosphide solution was added. Using ^31^P NMR spectroscopy, the concentration (9.097 mmol per 33 mL, 0.276 m) of the potassium phosphide solution was determined. ^31^P{^1^H} NMR (162 MHz, potassium bis(4‐dodecylphenyl)phosphide solution, DMSO‐*d*_6_ (internal capillary reference tube), 298 K): *δ*=−20.3 ppm (s, Ar_2_−*P*−K).

### Synthesis of bis{2‐[bis(4‐dodecylphenyl)phosphino]ethyl}‐amine (MACHO‐C_12_)

The synthesis was carried out adapting a literature procedure.^[12b]^ To a potassium bis(4‐dodecylphenyl)phosphide 2‐methyltetrahydrofuran/*n*‐pentane solution as described above (33 mL, 0.276 m, 9.097 mmol), a solution of potassium *tert*‐butoxide [0.571 g, 5.089 mmol, 1.1 equiv. referred to the necessary amount of bis(2‐chloroethyl)amine hydrochloride] in tetrahydrofuran (5 mL) was added. Next, bis(2‐chloroethyl)amine hydrochloride (0.820 g, 4.594 mmol, 0.505 equiv.) was added as a coarse, crystalline powder. The reaction mixture was heated to 60 °C and the reaction progress was monitored using ^31^P NMR spectroscopy (as unreacted potassium phosphide cannot be properly quantified in the ^31^P NMR spectrum, one drop of demineralized water was added into the NMR tube and the amount of unreacted potassium phosphide was determined indirectly by integrating the signal of the formed phosphine, which shows a typical doublet at around −40 ppm). After complete reaction, the solvent was removed under reduced pressure (1×10^−3^ mbar). The residue was dissolved in *n*‐heptane (30 mL) and the *n*‐heptane phase was extracted with demineralized water (3×10 mL) and methanol (3×10 mL) and then dried over MgSO_4_. The solvent was removed under reduced pressure (1×10^−3^ mbar) and the desired product was obtained as a dark brown, highly viscous oil (4.1 g, 3.66 mmol, 50 %). ^1^H NMR (400 MHz, methylene chloride‐*d*_2_, 298 K): *δ*=0.88 (t, ^3^ *J*_H,H_=6.8 Hz, 12H, −CH_2_−C*H*_3_), 1.27 (br. m, 72H, −CH_2_−C_9_ *H*_18_−CH_3_), 1.58 (m, 8H, −CH_2_−C*H*_2_−C_10_H_21_), 2.15 (t, ^3^ *J*_H,H_=7.6 Hz, 4H, N−CH_2_−C*H*_2_−P), 2.58 (t, ^3^ *J*_H,H_=7.6 Hz, 8H, Ar−C*H*_2_−C_11_H_23_), 2.66 (dt, ^3^ *J*_H,H_=7.6 Hz, ^3^ *J*_N‐H,H_=11.9 Hz, 4H, N−C*H*_2_−CH_2_−P), 7.14 (d, ^3^ *J*_H,H_=7.6 Hz, 8H, Ar−*H*), 7.30 ppm (dd, ^3^ *J*_H,H_=6.8 Hz, ^3^ *J*_P,H_=7.6 Hz, 8H, Ar−*H*). ^13^C NMR (101 MHz, methylene chloride‐*d*_2_, 298 K): *δ*=14.1 (s, −CH_2_−*C*H_3_), 22.7 (s, −*C*H_2_−), 29.2 (d, ^1^ *J*_C,P_=11.6 Hz, N−CH_2_−*C*H_2_−P), 29.4–29.7 (m, −*C*H_2_−), 31.3 (s, −CH_2_−*C*H_2_−C_10_H_21_), 32.0 (s, −*C*H_2_−), 35.8 (s, Ar−*C*H_2_−C_11_H_23_), 46.4 (d, ^2^ *J*_C,P_=20.9 Hz, N−*C*H_2_−CH_2_−P), 128.6 (d, ^3^ *J*_C,P_=6.8 Hz, Ar−*C*), 132.6 (d, ^2^ *J*_C,P_=18.9 Hz, Ar−*C*), 135.2 (d, ^1^ *J*_C,P_=11.1 Hz, P−*C*_q_), 143.5 ppm (s, Ar−*C*_q_). ^31^P{^1^H} NMR (162 MHz, methylene chloride‐*d*_2_, 298 K): *δ*=−23.0 ppm (s, Ar_2_−*P*−CH_2_). HRMS/ESI (pos): [C_76_H_126_NP_2_]^+^: calculated: 1114.93600 *m*/*z*. found: 1114.93635 *m*/*z*.

### Synthesis of [Ru(CO)ClH(MACHO‐C_12_)]

The synthesis was carried out adapting a literature procedure.^[13]^ In a 20 mL Schlenk tube, bis{2‐[bis(4‐dodecylphenyl)phosphino]ethyl}amine (2.978 g, 2.671 mmol, 1.0 equiv.) and [Ru(CO)ClH(P^*i*^Pr_3_)_2_] (1.168 g, 2.404 mmol, 0.90 equiv.) were dissolved in toluene (15 mL). The reaction mixture was heated to 110 °C and after 4 h reaction time a clear, orange solution was obtained. The solvent was removed under reduced pressure (1×10^−3^ mbar) and the residue was dissolvent in *n*‐heptane (30 mL). The *n*‐heptane phase was extracted with dimethyl sulfoxide (3×10 mL) to remove residues of ruthenium precursor. The solvent was removed under reduced pressure (1×10^−3^ mbar) and the desired product was obtained as dark orange viscous oil (3.3 g, 2.60 mmol, 98 %). ^1^H NMR (400 MHz, benzene‐*d*_6_, 298 K): *δ*=−14.76 (t, ^2^ *J*_H,P_=19.6 Hz, 1H, Ru−*H*), −13.88 (t, ^2^ *J*_H,P_=20.3 Hz, 1H, Ru−*H*), 0.92 (m, 12H, −CH_2_−C*H*_3_), 1.21–1.48 (br. m, 72H, −CH_2_−C_9_ *H*_18_−CH_3_), 2.04 (m, 2H, N−(C_2_ *H*_4_−P)_2_), 2.36–2.44 (m, 8H, Ar−C*H*_2_−C_11_H_23_), 2.51–2.56 (m, 4H, N−(C_2_ *H*_4_−P)_2_), 2.82–2.94 (m, 2H, N−(C_2_ *H*_4_−P)_2_), 4.77–4.82 (m, 1H, N−*H*), 5.48–5.51 (m, 1H, N−*H*), 6.96–7.13 (m, 8H, Ar−*H*), 7.86–8.21 ppm (m, 8H, Ar−*H*). ^13^C NMR (101 MHz, benzene‐*d*_6_, 298 K): *δ*=14.4 (s, −CH_2_−*C*H_3_), 23.2 (s, −*C*H_2_−), 29.8–30.3 (m, −*C*H_2_−), 31.6 (s, −*C*H_2_−), 31.8 (s, −*C*H_2_−), 32.4 (s, N−CH_2_−CH_2_−P), 36.1 (s, −*C*H_2_−), 36.3 (s, −*C*H_2_−), 52.4 (s, N−CH_2_−CH_2_−P), 128.6–128.8 (m, Ar−*C*), 132.8–134.5 (m, Ar−*C*), 144.7 ppm (d, ^1^ *J*_C,P_=22.3 Hz, P−*C*_q_). ^31^P{^1^H} NMR (162 MHz, benzene‐*d*_6_, 298 K): *δ*=50.9 (s, Ar_2_−*P*−CH_2_, *syn*‐isomer), 53.4 ppm (s, Ar_2_−*P*−CH_2_, *anti*‐isomer). Elemental analysis for C_77_H_126_ClNOP_2_Ru: calculated: C 72.24, H 9.92, N 1.09; found: C 71.29, H 9.71, N 1.17. HRMS/ESI (pos): [C_77_H_125_ClNOP_2_Ru]^+^: calculated: 1278.79739 *m*/*z*. found: 1278.79752 *m*/*z*. FT‐IT (ATR): $\tilde{\nu }$ =1914 cm^−1^ (s) (CO).

### General procedure for phase behavior experiments

Inside a glovebox, [Ru(CO)ClH(MACHO‐C_12_)] (53.9 mg, 42.1 μmol) was weighed into a 5 mL Schlenk tube and then dissolved in *n*‐decane (2.0 mL, 1.45 g). Next, an equimolar mixture of methanol (1.42 mL, 1.14 g, 0.035 mmol) and water (0.63 mL, 0.63 g, 0.035 mmol) was added to the solution and the resulting biphasic mixture was stirred vigorously for 5 min. Afterwards, both liquid phases were carefully separated via syringe and aliquots of both phases were analyzed by NMR spectroscopy (Figures S12–S17) and ICP‐MS.

### General procedure for DMC hydrogenation experiments

Inside a glovebox, [Ru(CO)ClH(MACHO‐C_12_)] and potassium *tert*‐butoxide were weighed into a 5 mL Schlenk tube. Next, *n*‐decane (2.5 mL) was added and the mixture was stirred for 1 h for deprotonation and activation of the Ru‐precursor (Figure S18). A 10 mL high pressure reactor with windows (Figure S19) equipped with a magnetic stirring bar, thermo element, and a digital pressure transducer was cycled with argon and vacuum (1×10^−3^ mbar) for at least three times. Under argon atmosphere, the reactor was loaded with the prepared deprotonated Ru‐MACHO‐C_12_ solution and DMC and sealed afterwards. The reactor was pressurized with H_2_, sealed, and placed onto a stirring plate with heating function. Heating of the stirring plate and recording of the pressure curve was started. After reaching 140 °C reaction temperature, stirring was started. After the reaction, the reactor was cooled to room temperature, vented, and a biphasic reaction mixture was obtained. Both liquid phases were carefully separated via syringe and the catalyst phase was diluted with acetone (3.0 mL). Mesitylene (internal standard) was added to both phases and aliquots of both phases were analyzed by NMR spectroscopy (Figures S20–S23). When 10 mmol DMC was used, the methanol amount was insufficient to allow for an appropriate separation of both liquid phases. Therefore, the biphasic reaction mixture obtained after the reaction was homogenized with acetone (3.0 mL) in the reactor and an aliquot of the resulting monophasic reaction mixture was analyzed by NMR spectroscopy.

### General procedure for CO_2_ hydrogenation experiments

Inside a glovebox, [Ru(CO)ClH(MACHO‐C_12_)] (6.4 mg, 5.0 μmol) and potassium *tert*‐butoxide (1.10 mg, 0.01 mmol) were weighed into a 5 mL Schlenk tube. Next, *n*–decane (2.5 mL) was added and the mixture was stirred for 1 h for deprotonation and activation of the Ru‐precursor (Figure S18). A 10 mL high pressure reactor with or without windows (Figures S19 and S24) equipped with a magnetic stirring bar, thermo element, and a digital pressure transducer was cycled with argon and vacuum (1×10^−3^ mbar) for at least three times. Under argon atmosphere, the reactor was loaded with the prepared deprotonated Ru‐MACHO‐C_12_ solution and *N,N′*‐dimethylethane‐1,2‐diamine (0.882 g, 10.0 mmol, 1.076 mL) and sealed afterwards. In order to saturate the reaction solution with carbon dioxide, the reactor was pressurized with carbon dioxide (20 bar) at room temperature for 10 min while stirring. After 10 min the overpressure was released, water (0.4 mL) was added (if due), and the reactor pressurized with H_2_ (200 bar). The reactor was sealed and placed onto a stirring plate with heating function. Heating of the stirring plate and recording of the pressure curve was started. After reaching 145 °C reaction temperature, stirring was started. After 48 h reaction time, the reactor was cooled to room temperature, vented, and a biphasic reaction mixture was obtained. Both liquid phases were carefully separated via syringe and diluted with *iso*‐propanol (3.0 mL). Internal standard was added (1,4‐dioxane for product phase, mesitylene for catalyst phase) and aliquots of both phases were analyzed by NMR spectroscopy (Figures S25–S28).

### Catalyst recycling

The first hydrogenation experiment was performed as described above in a 10 mL high pressure reactor with windows using [Ru(CO)ClH(MACHO‐C_12_)] (6.40 mg, 5.0 μmol), potassium *tert*‐butoxide (10 μmol, 1.1 mg), and *N,N′*‐dimethylethane‐1,2‐diamine (0.882 g, 10.0 mmol, 1.076 mL). After 48 h reaction time the reactor was cooled to room temperature, vented to around 1 bar, and the remaining 1 bar was released carefully through the Schlenk line with overpressure bubbler under argon atmosphere. The product phase was carefully removed via syringe without removing the catalyst phase (as the needle should pass through the upper *n*‐decane catalyst phase to reach the bottom product phase, very slight cross contamination of the product phase cannot be excluded) and diluted with *iso*‐propanol (3.0 mL). 1,4‐Dioxane (internal standard) was added and an aliquot was analyzed by NMR spectroscopy and ICP‐MS. The reactor was loaded again with the same amount of *N,N′*‐dimethylethane‐1,2‐diamine, and the procedure was repeated. After the last recycling run, the catalyst phase was analyzed as well by NMR spectroscopy (Tables S1 and S2).

When working in standard high pressure reactors without windows, the product and catalyst phase were separated in a syringe in argon counterflow so that both phases remained under argon atmosphere for the whole process. The catalyst phase was then returned to the reactor while the polar phase was removed and treated as described above (Table S3).

## Conflict of interest

The authors declare no conflict of interest.

1

## Supporting information

As a service to our authors and readers, this journal provides supporting information supplied by the authors. Such materials are peer reviewed and may be re‐organized for online delivery, but are not copy‐edited or typeset. Technical support issues arising from supporting information (other than missing files) should be addressed to the authors.

Supporting Information

## Data Availability

The data that support the findings of this study are available in the supplementary material of this article.

## References

[1a] A. Behr , Angew. Chem. Int. Ed. 1988, 27, 661–678;

[1b] W. Leitner , Angew. Chem. Int. Ed. 1995, 34, 2207–2221;

[1c] P. G. Jessop , T. Ikariya , R. Noyori , Chem. Rev. 1995, 95, 259–272;

[1d] Y. Himeda , in CO_2_ Hydrogenation Catalysis, Wiley-VCH, Weinheim, 2021.

[2a] M. Cokoja , C. Bruckmeier , B. Rieger , W. A. Herrmann , F. E. Kühn , Angew. Chem. Int. Ed. 2011, 50, 8510–8537; 10.1002/anie.201102010 21887758

[2b] M. Peters , B. Köhler , W. Kuckshinrichs , W. Leitner , P. Markewitz , T. E. Müller , ChemSusChem 2011, 4, 1216–1240; 21866580 10.1002/cssc.201000447

[2c] M. Aresta , A. Dibenedetto , A. Angelini , Chem. Rev. 2014, 114, 1709–1742; 24313306 10.1021/cr4002758

[2d] J. Klankermayer , W. Leitner , Science 2015, 350, 629–630; 26542554 10.1126/science.aac7997

[2e] W.-H. Wang , Y. Himeda , J. T. Muckerman , G. F. Manbeck , E. Fujita , Chem. Rev. 2015, 115, 12936–12973; 26335851 10.1021/acs.chemrev.5b00197

[2f] J. B. Zimmerman , P. T. Anastas , H. C. Erythropel , W. Leitner , Science 2020, 367, 397–400. 31974246 10.1126/science.aay3060

[3a] G. Centi , E. A. Quadrelli , S. Perathoner , Energy Environ. Sci. 2013, 6, 1711–1731;

[3b] J. Artz , T. E. Müller , K. Thenert , J. Kleinekorte , R. Meys , A. Sternberg , A. Bardow , W. Leitner , Chem. Rev. 2018, 118, 434–504; 29220170 10.1021/acs.chemrev.7b00435

[3c] R. Schlögl , Angew. Chem. Int. Ed. 2022, 61, e202007397. 10.1002/anie.202007397 PMC9299727 32816338

[4a] F. Asinger , in Methanol – Chemie- und Energierohstoff, Springer-Verlag, Berlin Heidelberg, 1986;

[4b] G. A. Olah , Angew. Chem. Int. Ed. 2005, 44, 2636–2639; 10.1002/anie.200462121 15800867

[4c] G. A. Olah , G. K. S. Prakash , A. Goeppert , J. Am. Chem. Soc. 2011, 133, 12881–12898; 21612273 10.1021/ja202642y

[4d] M. Bertau , H. Offermanns , L. Plass , F. Schmidt , H.-J. Wernicke , in Methanol: The Basic Chemical and Energy Feedstock of the Future, Springer-Verlag, Berlin Heidelberg, 2014;

[4e] G. A. Olah , A. Goeppert , G. K. S. Prakash , in Beyond Oil and Gas: The Methanol Economy, Wiley-VCH, Weinheim, 2018;

[4f] S.-T. Bai , G. De Smet , Y. Liao , R. Sun , C. Zhou , M. Beller , B. U. W. Maes , B. F. Sels , Chem. Soc. Rev. 2021, 50, 4259-4298; 33687387 10.1039/d0cs01331e

[4g] S. Kar , A. Goeppert , G. K. Surya Prakash , in CO_2_ Hydrogenation Catalysis, Ed. Y. Himeda , Wiley-VCH, Weinheim, 2021, pp. 89–112.

[5a] J. Klankermayer , S. Wesselbaum , K. Beydoun , W. Leitner , Angew. Chem. Int. Ed. 2016, 55, 7296–7343; 10.1002/anie.201507458 27237963

[5b] K. Sordakis , C. Tang , L. K. Vogt , H. Junge , P. J. Dyson , M. Beller , G. Laurenczy , Chem. Rev. 2018, 118, 372–433. 28985048 10.1021/acs.chemrev.7b00182

[6a] E. Balaraman , C. Gunanathan , J. Zhang , L. J. W. Shimon , D. Milstein , Nat. Chem. 2011, 3, 609; 21778980 10.1038/nchem.1089

[6b] E. Balaraman , Y. Ben-David , D. Milstein , Angew. Chem. Int. Ed. 2011, 50, 11702–11705; 10.1002/anie.201106612 22052711

[6c] C. A. Huff , M. S. Sanford , J. Am. Chem. Soc. 2011, 133, 18122–18125; 22029268 10.1021/ja208760j

[6d] S. Wesselbaum , T. vom Stein , J. Klankermayer , W. Leitner , Angew. Chem. Int. Ed. 2012, 51, 7499–7502; 10.1002/anie.201202320 22707348

[6e] S. Wesselbaum , V. Moha , M. Meuresch , S. Brosinski , K. M. Thenert , J. Kothe , T. v. Stein , U. Englert , M. Holscher , J. Klankermayer , W. Leitner , Chem. Sci. 2015, 6, 693–704; 30154993 10.1039/c4sc02087a PMC6085670

[6f] B. G. Schieweck , P. Jürling-Will , J. Klankermayer , ACS Catal. 2020, 3890–3894.

[7] Z. Han , L. Rong , J. Wu , L. Zhang , Z. Wang , K. Ding , Angew. Chem. Int. Ed. 2012, 51, 13041–13045; 10.1002/anie.201207781 23161665

[8a] N. M. Rezayee , C. A. Huff , M. S. Sanford , J. Am. Chem. Soc. 2015, 137, 1028–1031; 25594380 10.1021/ja511329m

[8b] J. Kothandaraman , A. Goeppert , M. Czaun , G. A. Olah , G. K. S. Prakash , J. Am. Chem. Soc. 2016, 138, 778–781; 26713663 10.1021/jacs.5b12354

[8c] S. Kar , R. Sen , A. Goeppert , G. K. S. Prakash , J. Am. Chem. Soc. 2018, 140, 1580–1583; 29363957 10.1021/jacs.7b12183

[8d] S. Kar , R. Sen , J. Kothandaraman , A. Goeppert , R. Chowdhury , S. B. Munoz , R. Haiges , G. K. S. Prakash , J. Am. Chem. Soc. 2019, 141, 3160–3170; 30753062 10.1021/jacs.8b12763

[8e] S. Kar , A. Goeppert , G. K. S. Prakash , ChemSusChem 2019, 12, 3172–3177. 30859718 10.1002/cssc.201900324

[9a] M. Scott , B. Blas Molinos , C. Westhues , G. Franciò , W. Leitner , ChemSusChem 2017, 10, 1085–1093; 28103428 10.1002/cssc.201601814 PMC5396146

[9b] W. Leitner , G. Franciò , M. Scott , C. Westhues , J. Langanke , M. Lansing , C. Hussong , E. Erdkamp , Chem. Ing. Tech. 2018, 90, 1504–1512;

[9c] C. M. Jens , M. Scott , B. Liebergesell , C. G. Westhues , P. Schäfer , G. Franciò , K. Leonhard , W. Leitner , A. Bardow , Adv. Synth. Catal. 2019, 361, 307–316;

[9d] M. Scott , C. G. Westhues , T. Kaiser , J. C. Baums , A. Jupke , G. Franciò , W. Leitner , Green Chem. 2019, 21, 6307–6317.

[10] Q. Wang , L. Wang , L. Yu , J. Am. Chem. Soc. 1998, 120, 12860–12868.

[11a] A. Bollmann , K. Blann , J. T. Dixon , F. M. Hess , E. Killian , H. Maumela , D. S. McGuinness , D. H. Morgan , A. Neveling , S. Otto , M. Overett , A. M. Z. Slawin , P. Wasserscheid , S. Kuhlmann , J. Am. Chem. Soc. 2004, 126, 14712–14713; 15535683 10.1021/ja045602n

[11b] S. Schweizer , J.-M. Becht , C. Le Drian , Adv. Synth. Catal. 2007, 349, 1150–1158;

[11c] L. E. Bowen , M. Charernsuk , T. W. Hey , C. L. McMullin , A. G. Orpen , D. F. Wass , Dalton Trans. 2010, 39, 560–567; 10.1039/b913302j 20023994

[11d] B. M. Trost , D. J. Michaelis , J. Charpentier , J. Xu , Angew. Chem. Int. Ed. 2012, 51, 204–208; 10.1002/anie.201105801 PMC3517038 22095749

[11e] D. Müller , L. Guénée , A. Alexakis , Eur. J. Org. Chem. 2013, 2013, 6335–6343.

[12a] H. Niebergall , B. Langenfeld , Chem. Ber. 1962, 95, 64–76;

[12b] R. G. Nuzzo , S. L. Haynie , M. E. Wilson , G. M. Whitesides , J. Org. Chem. 1981, 46, 2861–2867.

[13] W. Kuriyama , T. Matsumoto , O. Ogata , Y. Ino , K. Aoki , S. Tanaka , K. Ishida , T. Kobayashi , N. Sayo , T. Saito , Org. Process Res. Dev. 2012, 16, 166–171.

[14] M. Tamizmani , C. Sivasankar , J. Organomet. Chem. 2017, 845, 82–89.

[15] C. Gunanathan , D. Milstein , Chem. Rev. 2014, 114, 12024–12087. 25398045 10.1021/cr5002782

[16] S. G. Prakash , R. Sen , A. Goeppert , Angew. Chem. Int. Ed. 2022, doi.org/10.1002/anie.202207278. 10.1002/anie.202207278 PMC9825957 35921247

[17] M. Everett , D. F. Wass , Chem. Commun. 2017, 53, 9502–9504. 10.1039/c7cc04613h 28661540

[18] F.-H. Zhang , C. Liu , W. Li , G.-L. Tian , J.-H. Xie , Q.-L. Zhou , Chin. J. Chem. 2018, 36, 1000–1002.

[19] For a recent example see: S. Püschel , J. Sadowski , T. Rösler , K. R. Ehmann , A. J. Vorholt , W. Leitner , ACS Sustainable Chem. Eng. 2022, 10, 3749–3756. 35360052 10.1021/acssuschemeng.2c00419 PMC8942186

[20] M. A. Esteruelas , H. Werner , J. Organomet. Chem. 1986, 303, 221–231.

[21a] G. R. Fulmer , A. J. M. Miller , N. H. Sherden , H. E. Gottlieb , A. Nudelman , B. M. Stoltz , J. E. Bercaw , K. I. Goldberg , Organometallics 2010, 29, 2176–2179;

[21b] N. R. Babij , E. O. McCusker , G. T. Whiteker , B. Canturk , N. Choy , L. C. Creemer , C. V. D. Amicis , N. M. Hewlett , P. L. Johnson , J. A. Knobelsdorf , F. Li , B. A. Lorsbach , B. M. Nugent , S. J. Ryan , M. R. Smith , Q. Yang , Org. Process Res. Dev. 2016, 20, 661–667.

