# Supplementary material for: A Self‐Separating Multiphasic System for Catalytic Hydrogenation of CO2 and CO2‐Derivatives to Methanol
Source: ChemSusChem. 2022 Oct 26;15(22):e202201250. doi: 10.1002/cssc.202201250 (PMC9828205; doi:10.1002/cssc.202201250)
Supplement: Supplementary file 1 — Supporting Information [file CSSC-15-0-s001.pdf]

ChemSusChem

Supporting Information

A Self-Separating Multiphasic System for Catalytic Hydrogenation of CO_2 and CO_2-Derivatives to Methanol

Thomas Diehl, Patrick Lanzerath, Giancarlo Franciò,* and Walter Leitner*© 2022 The Authors. ChemSusChem published by Wiley-VCH GmbH. This is an open access article under the terms of the Creative Commons Attribution License, which permits use, distribution and reproduction in any medium, provided the original work is properly cited.

Supporting Information
©Wiley-VCH 2021
69451 Weinheim, Germany

A Self-Separating Multiphasic System for Catalytic Hydrogenation of CO_2 and CO_2-Derivatives to Methanol

Thomas Diehl, Patrick Lanzerath, Giancarlo Franciò,* and Walter Leitner*

Abstract: Catalytic conversion of CO_2 and hydrogen to methanol was achieved in a self-separating multiphasic system comprising the tailor-made complex [Ru(CO)ClH(MACHO-C_{12})] (MACHO-C_{12} = bis{2-[bis(4-dodecylphenyl)phosphino]ethyl}amine) in *n*-decane as the catalyst phase. Effective catalyst recycling was demonstrated for the carbonate and the amine-assisted pathway from CO_2 to methanol. The polar products MeOH or MeOH/H_2O generated from the catalytic reactions form spontaneously a separate phase allowing product isolation and catalyst separation without the need for any additional solvent. In the amine-assisted hydrogenation of CO_2, the catalyst phase was recycled over ten subsequent runs reaching a total turnover number (TTON) to MeOH of 19.200 with an average selectivity of 96%.

DOI: 10.1002/anie.2021XXXXX

Contents

1. Experimental 3

1.1 NMR spectra of chloro-bis(4-dodecylphenyl)phosphine 3

1.2 NMR spectra of potassium bis(4-dodecylphenyl)phosphide 5

1.3 NMR spectra of bis{2-[bis(4-dodecylphenyl)phosphino]ethyl}amine (MACHO-C_{12}) 6

1.4 NMR spectra and IR spectrum of [Ru(CO)ClH(MACHO-C_{12})] 8

1.5 Phase behavior experiments 11

1.6 DMC hydrogenation experiments 15

1.7 CO_2 hydrogenation experiments 18

1.8 Catalyst Recycling 21

1. Experimental

1.1 NMR spectra of chloro-bis(4-dodecylphenyl)phosphine

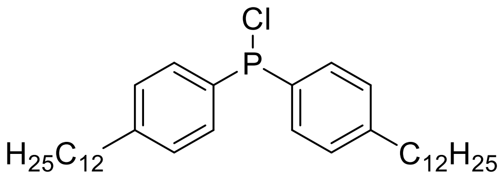

$C_{36}H_{58}ClP$

557.28 g/mol

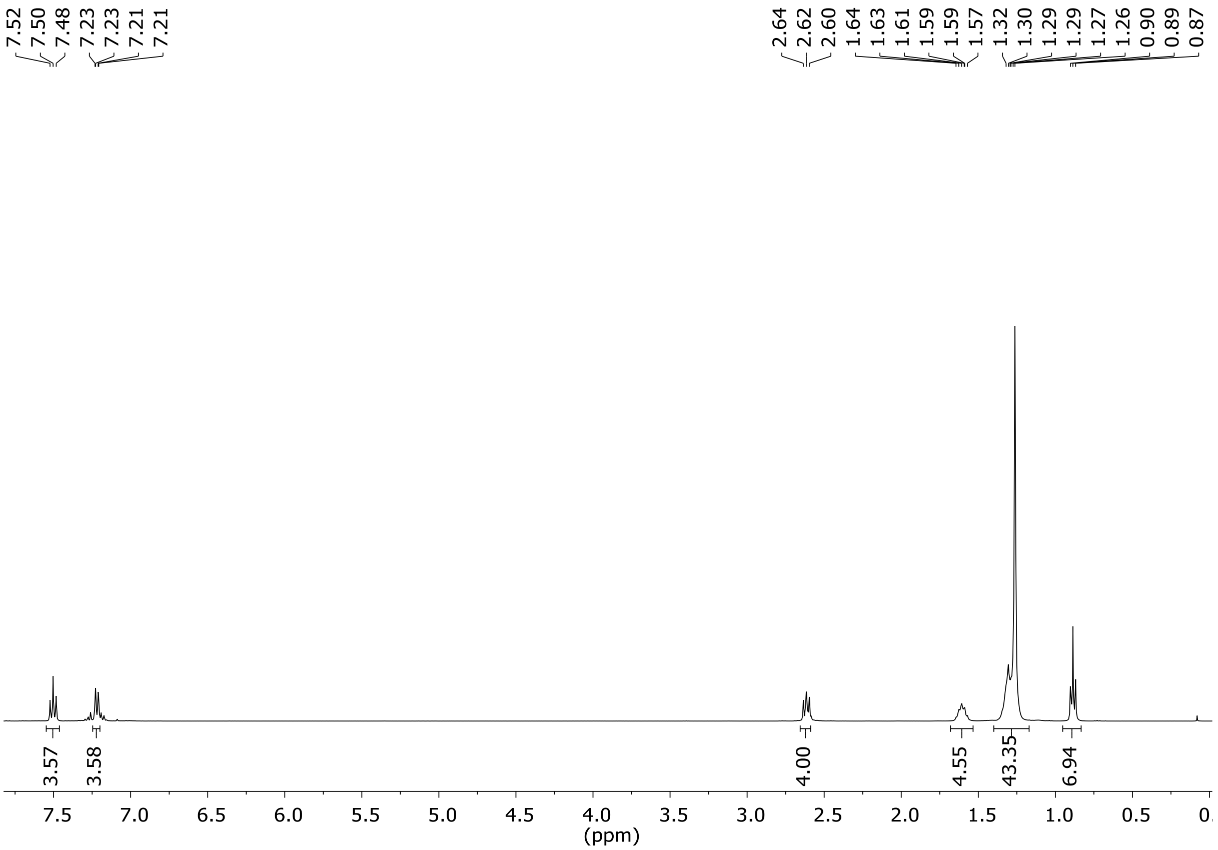

Figure S1. ^{1}H-NMR (400 MHz, chloroform-*d*, 298 K) for chloro-bis(4-dodecylphenyl)phosphine.

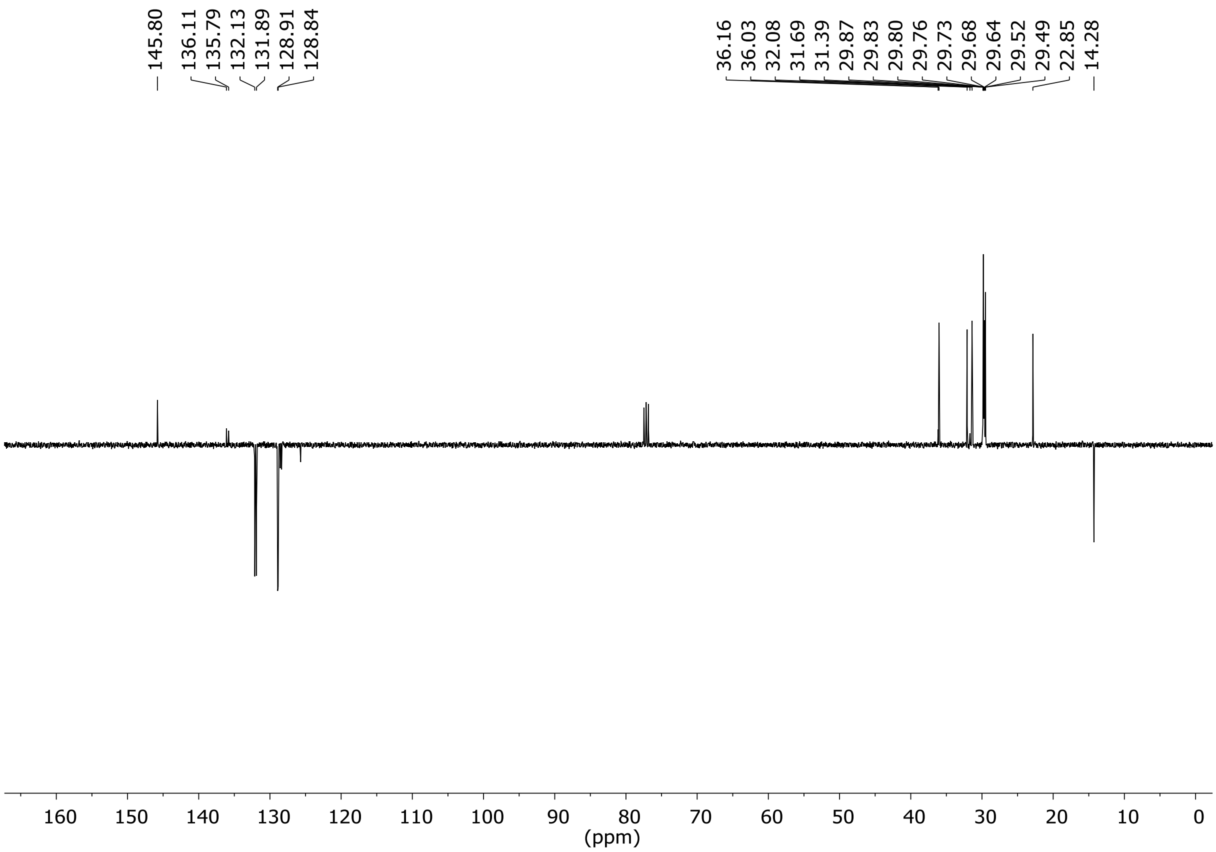

Figure S2. ^{13}C-NMR (101 MHz, chloroform-*d*, 298 K) for chloro-bis(4-dodecylphenyl)phosphine.

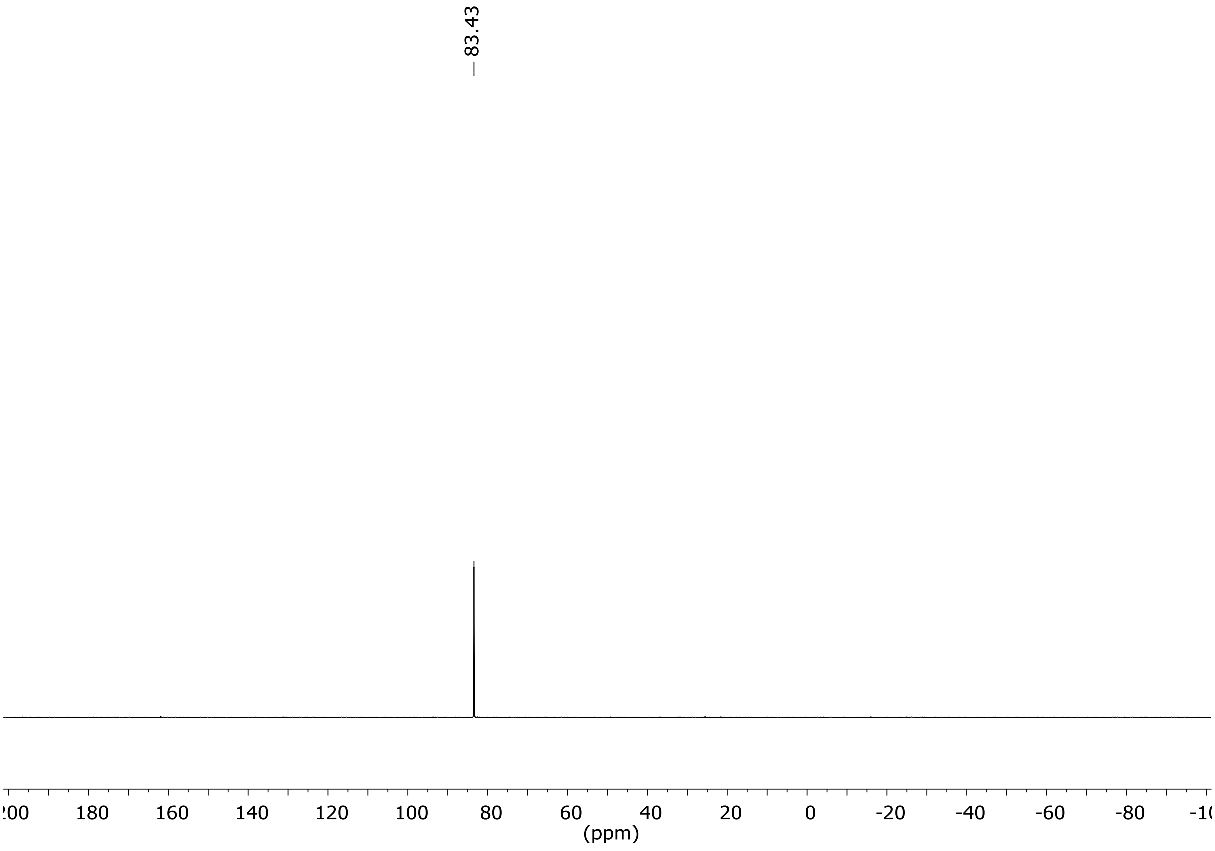

Figure S3. ^{31}P{^{1}H}-NMR (162 MHz, chloroform-*d*, 298 K) for chloro-bis(4-dodecylphenyl)phosphine.

1.2 NMR spectra of potassium bis(4-dodecylphenyl)phosphide

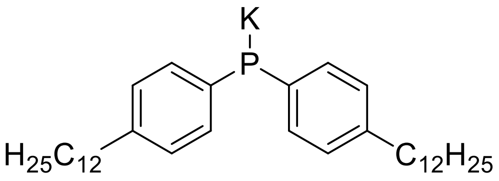

$C_{36}H_{58}KP$

560.93 g/mol

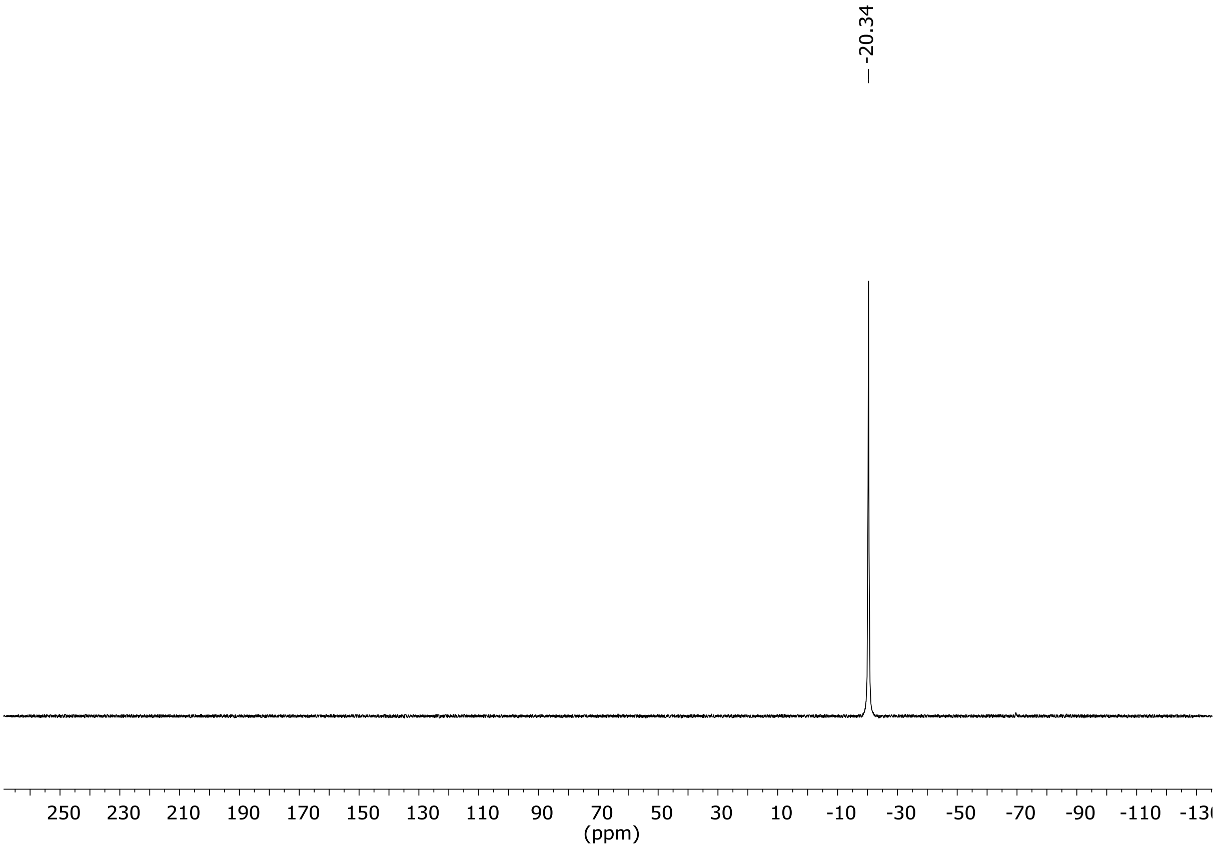

Figure S4. $^{31}P\{^{1}H\}$-NMR (162 MHz, dmso-d_6 *(capillary tube)*, 298 K) for potassium bis(4-dodecylphenyl)phosphide.

1.3 NMR spectra of bis{2-[bis(4-dodecylphenyl)phosphino]ethyl}amine (MACHO-C_{12})

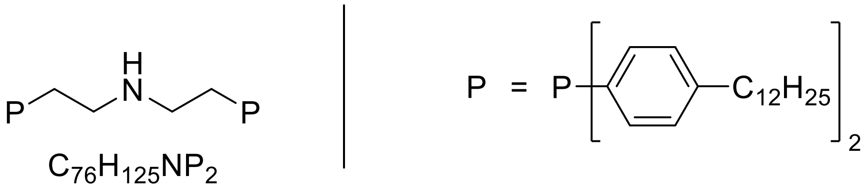

$C_{76}H_{125}NP_2$

1114.79 g/mol

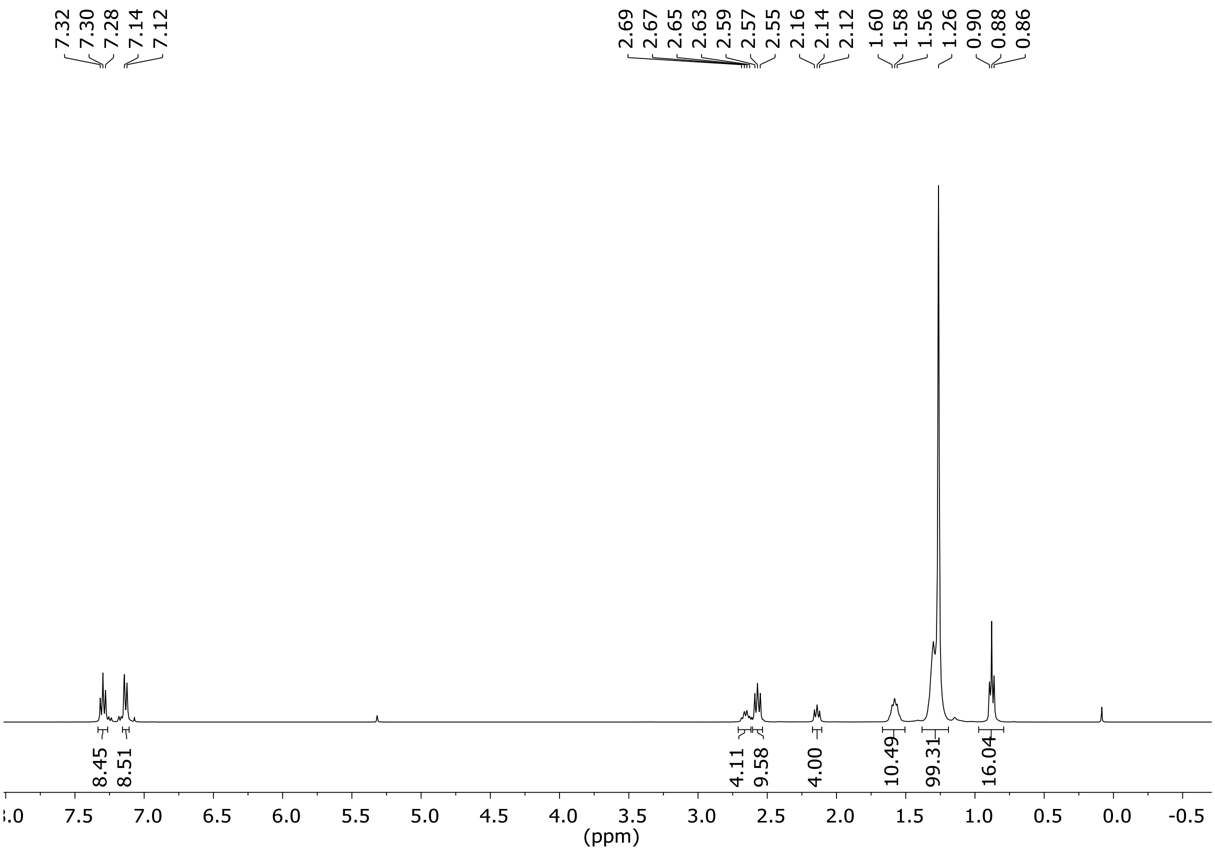

Figure S5. ^{1}H-NMR (400 MHz, Methylene chloride-d_2, 298 K) for bis{2-[bis(4-dodecylphenyl)phosphino]ethyl}amine.

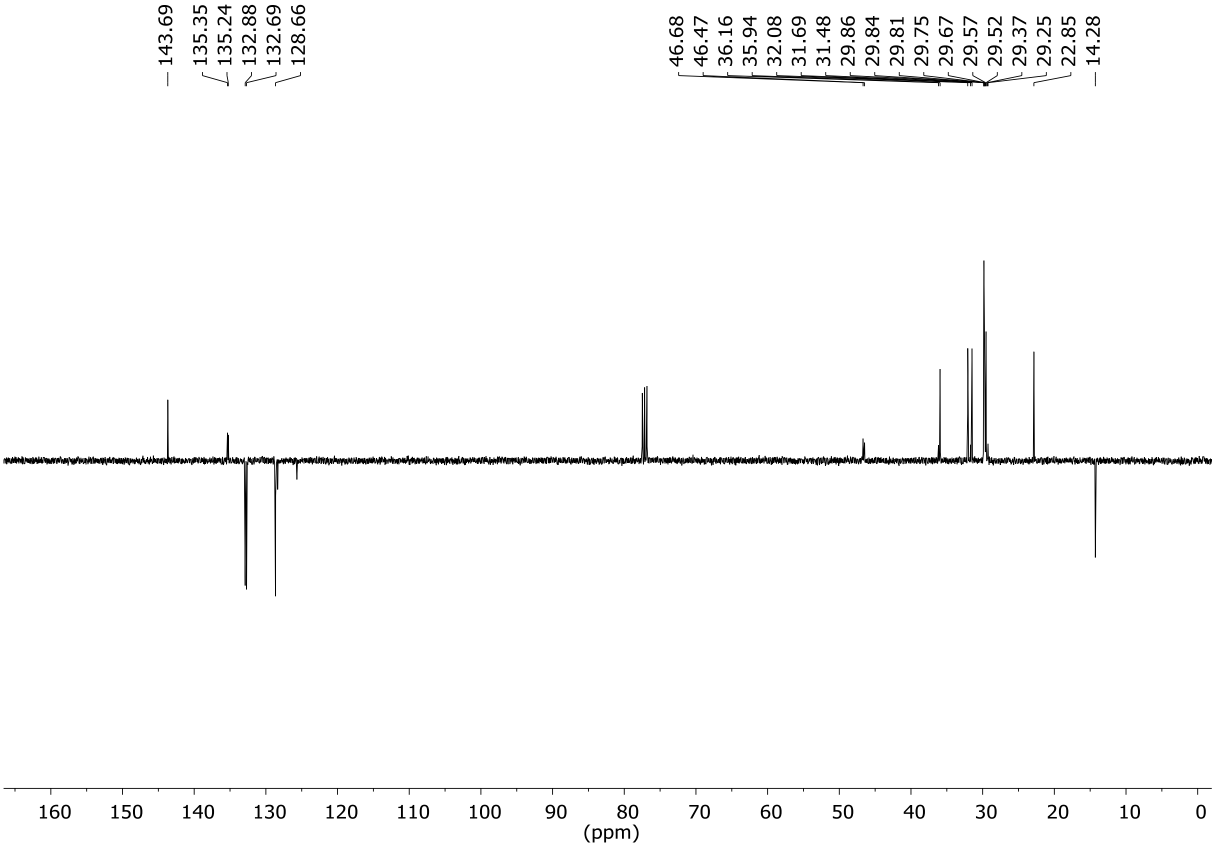

Figure S6. ^{13}C-NMR (162 MHz, chloroform-*d*, 298 K) for bis{2-[bis(4-dodecylphenyl)phosphino]ethyl}amine.

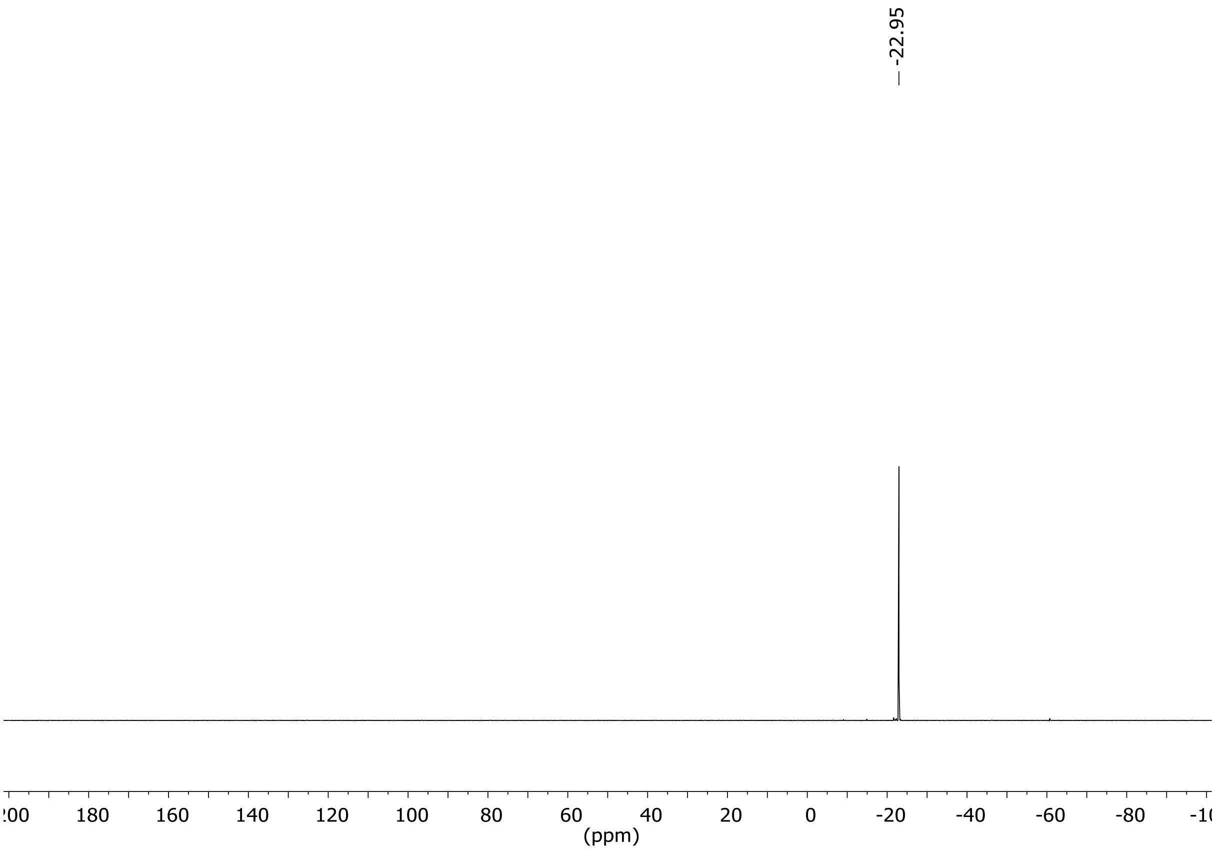

Figure S7. $^{31}P\{^{1}H\}$-NMR (162 MHz, Methylene chloride-d_2, 298 K) for bis{2-[bis(4-dodecylphenyl)phosphino]ethyl}amine.

1.4 NMR spectra and IR spectrum of [Ru(CO)ClH(MACHO-C12)]

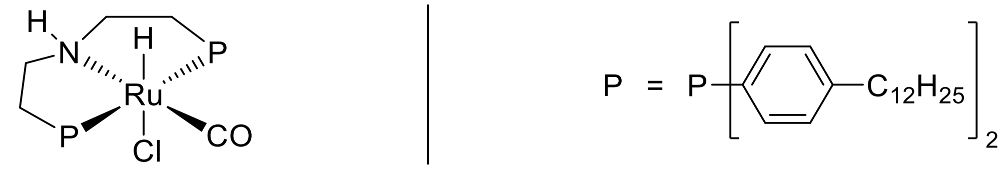

$C_{77}H_{126}ClNOP_2Ru$

1280.33 g/mol

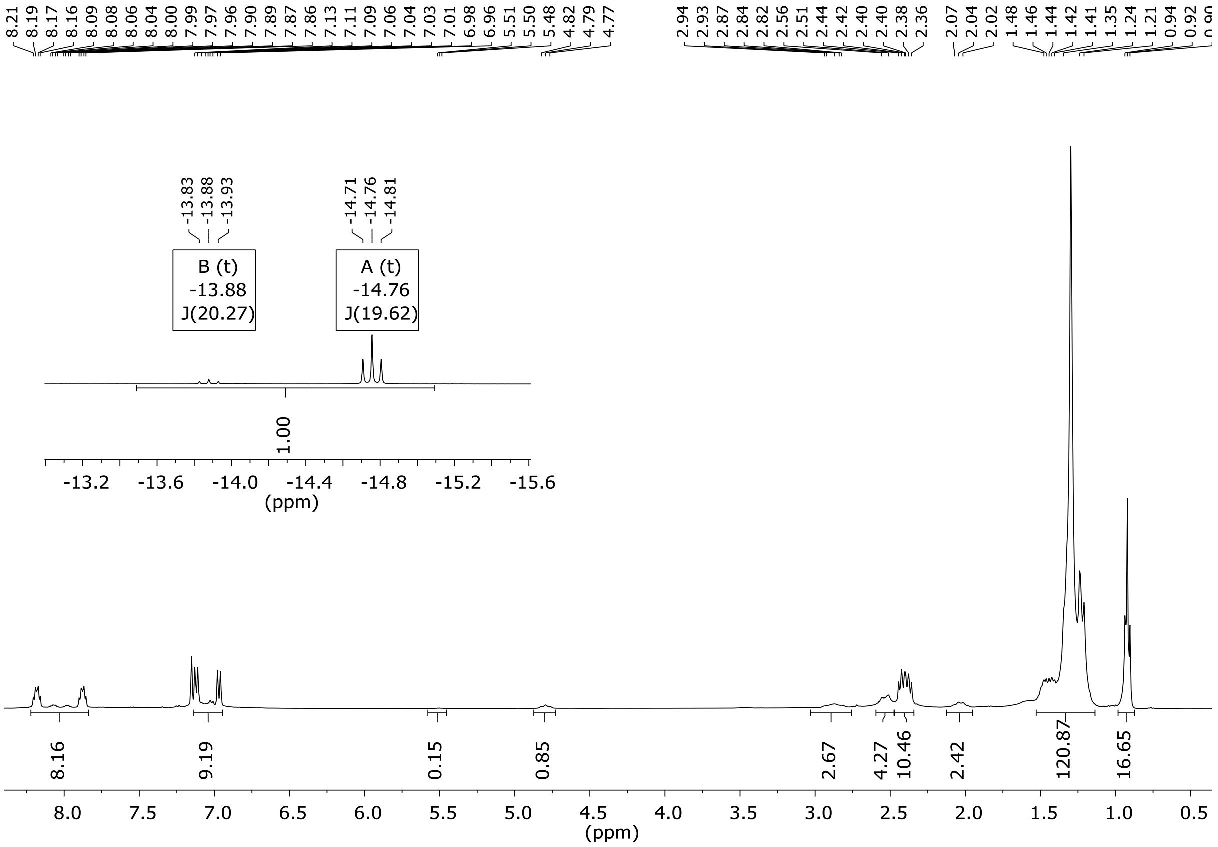

Figure S8. ^{1}H-NMR (400 MHz, benzene-d_6, 298 K) for [Ru(CO)ClH(MACHO-C12)].

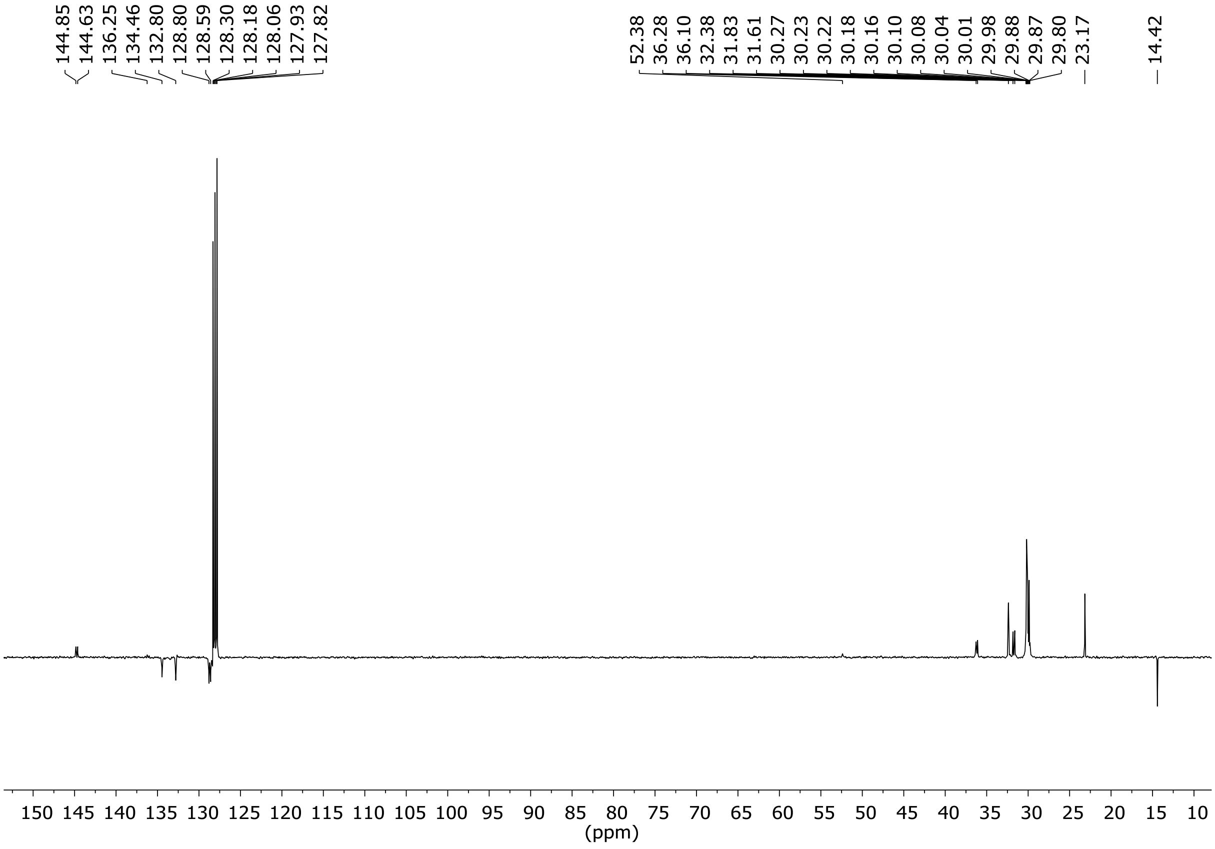

Figure S9. ^{13}C-NMR (101 MHz, benzene-d_6, 298 K) for [Ru(CO)ClH(MACHO-C_{12})].

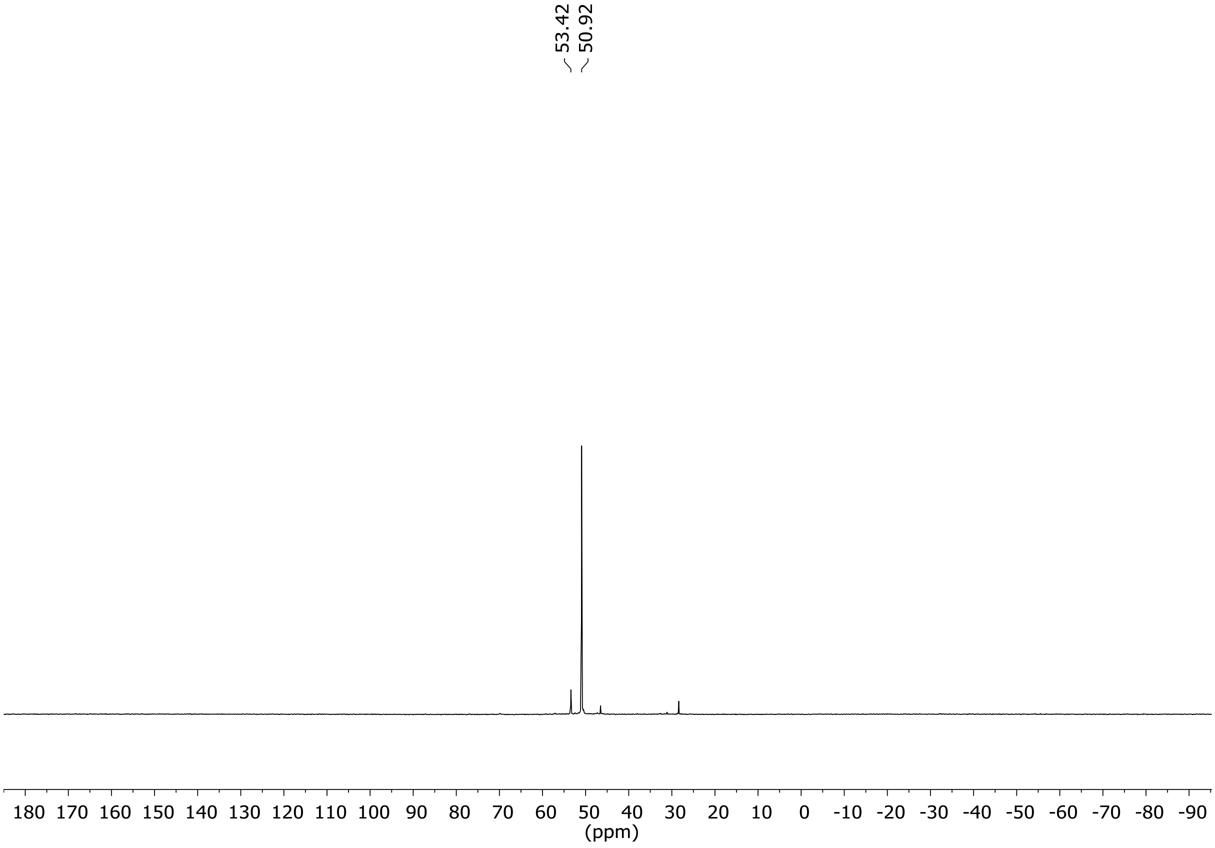

Figure S10. $^{31}P\{^1H\}$-NMR (162 MHz, benzene-d_6, 298 K) for [Ru(CO)ClH(MACHO-C_{12})].

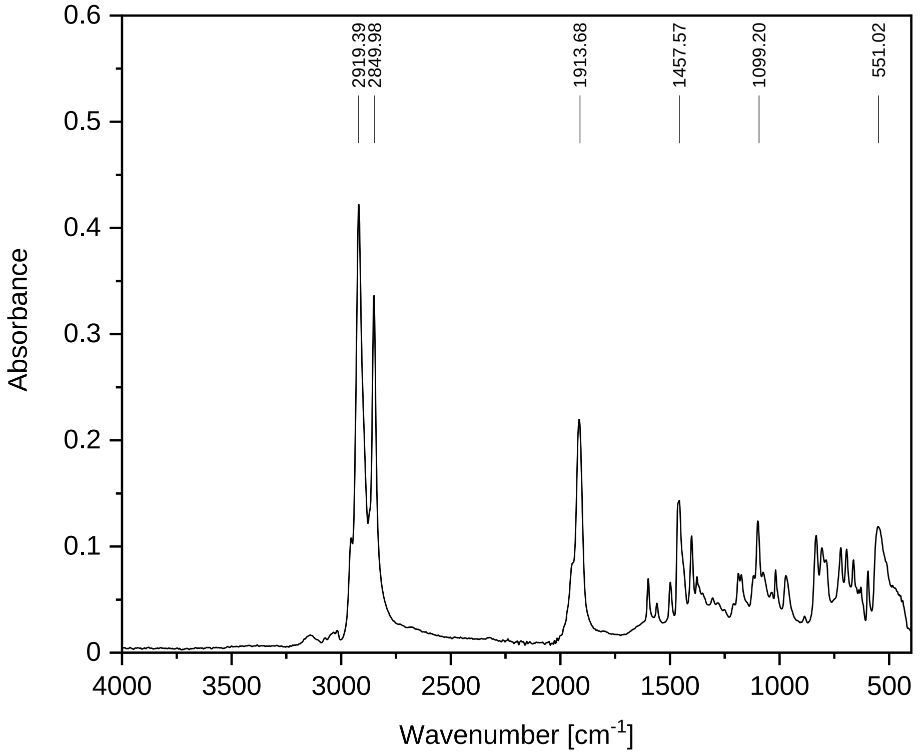

Figure S11. ATR-IR-spectrum for [Ru(CO)ClH (MACHO-C_{12})].

1.5 Phase behavior experiments

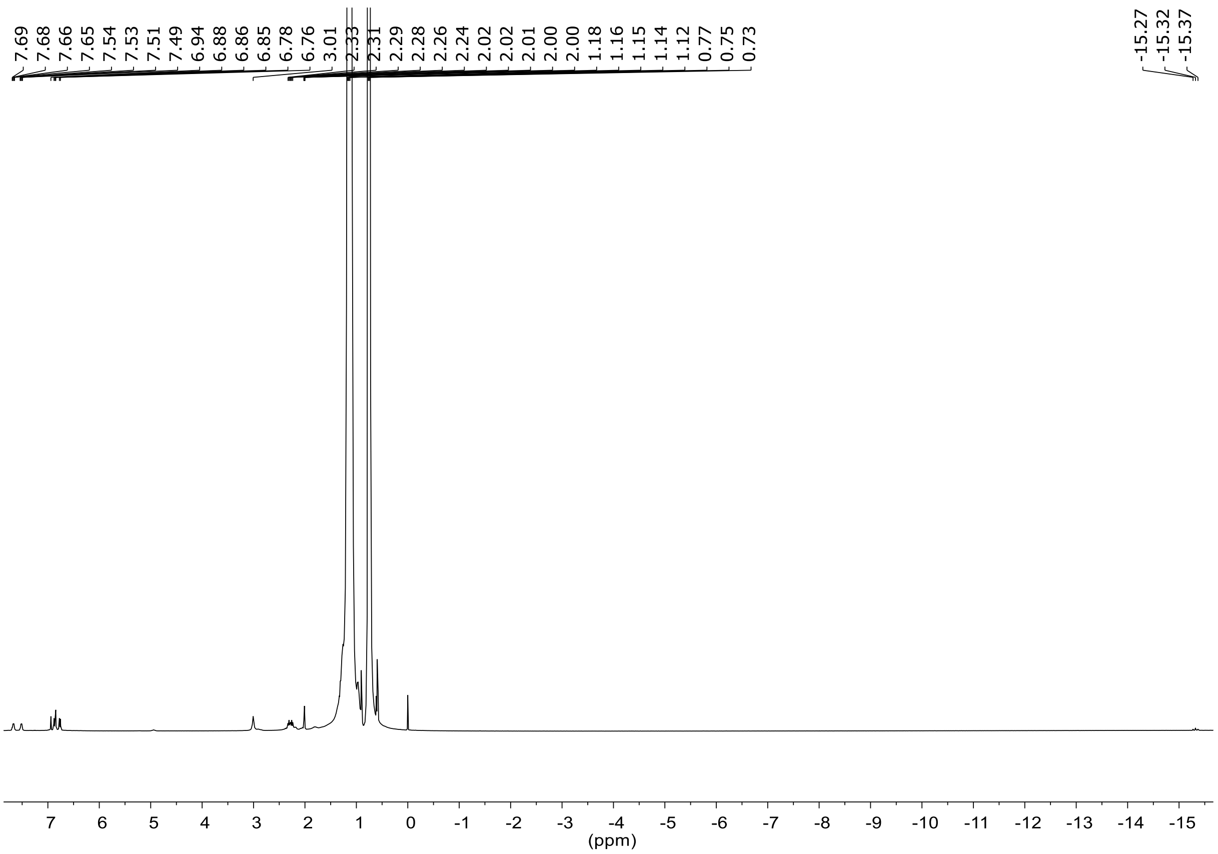

Figure S12. ^{1}H-NMR spectrum (400 MHz, chloroform-*d*) of the nonpolar *n*-decane phase.

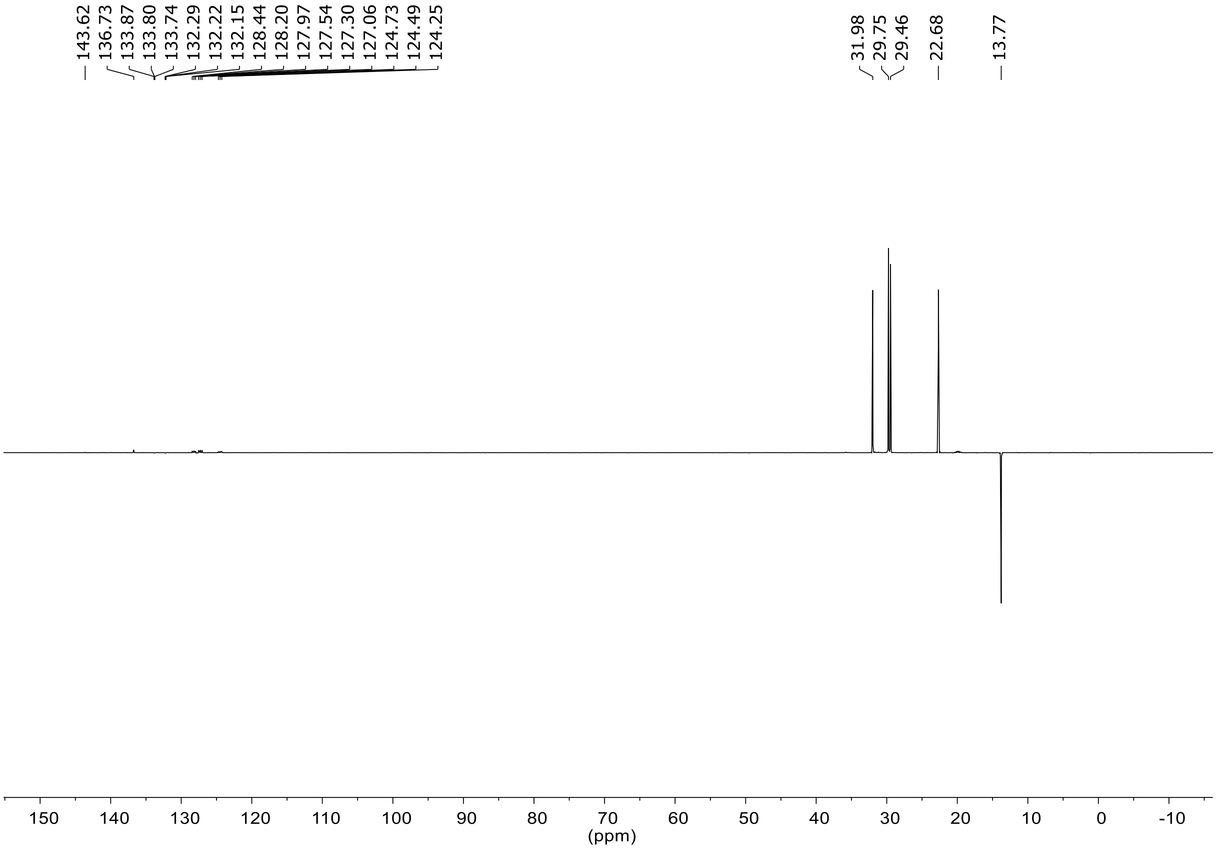

Figure S13. ^{13}C-NMR spectrum (101 MHz, chloroform-*d*) of the nonpolar *n*-decane phase.

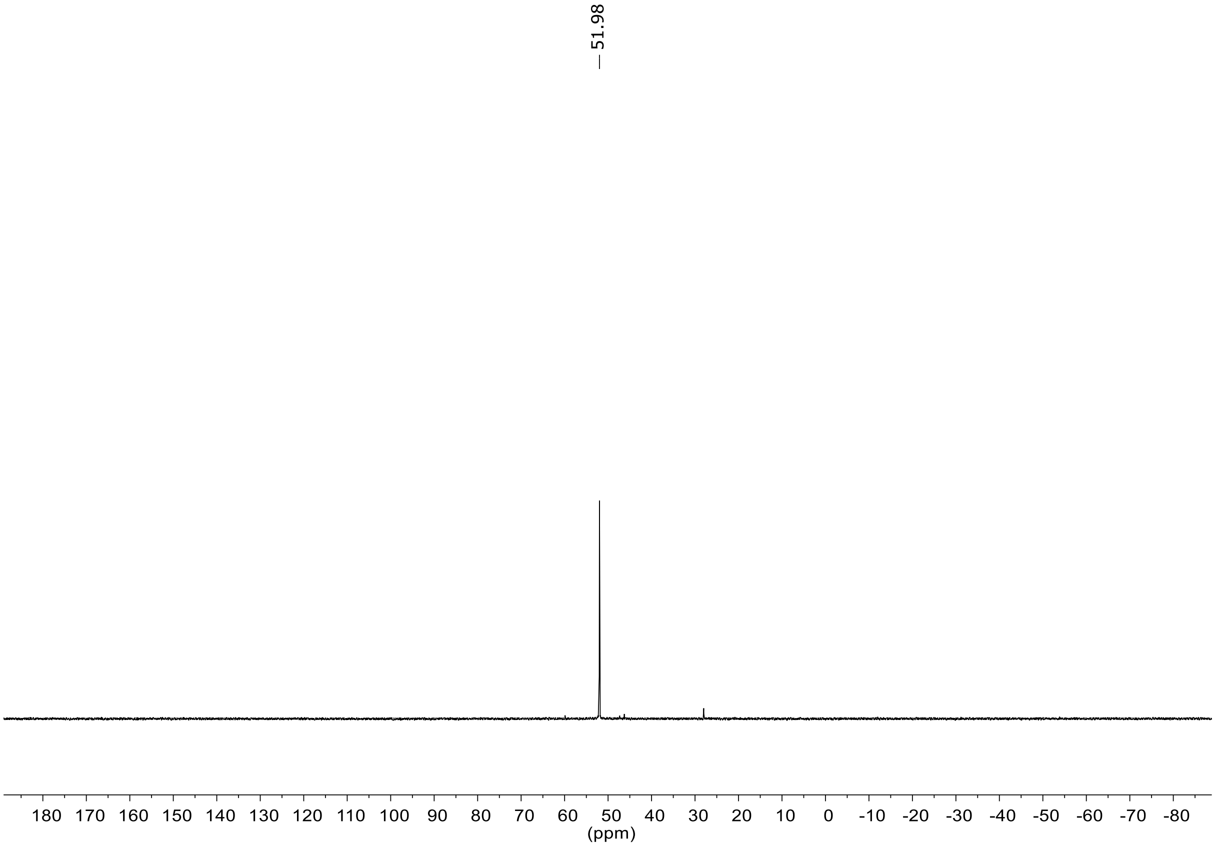

Figure S14. ^{31}P{^{1}H}-NMR spectrum (162 MHz, chloroform-*d*) of the nonpolar *n*-decane phase.

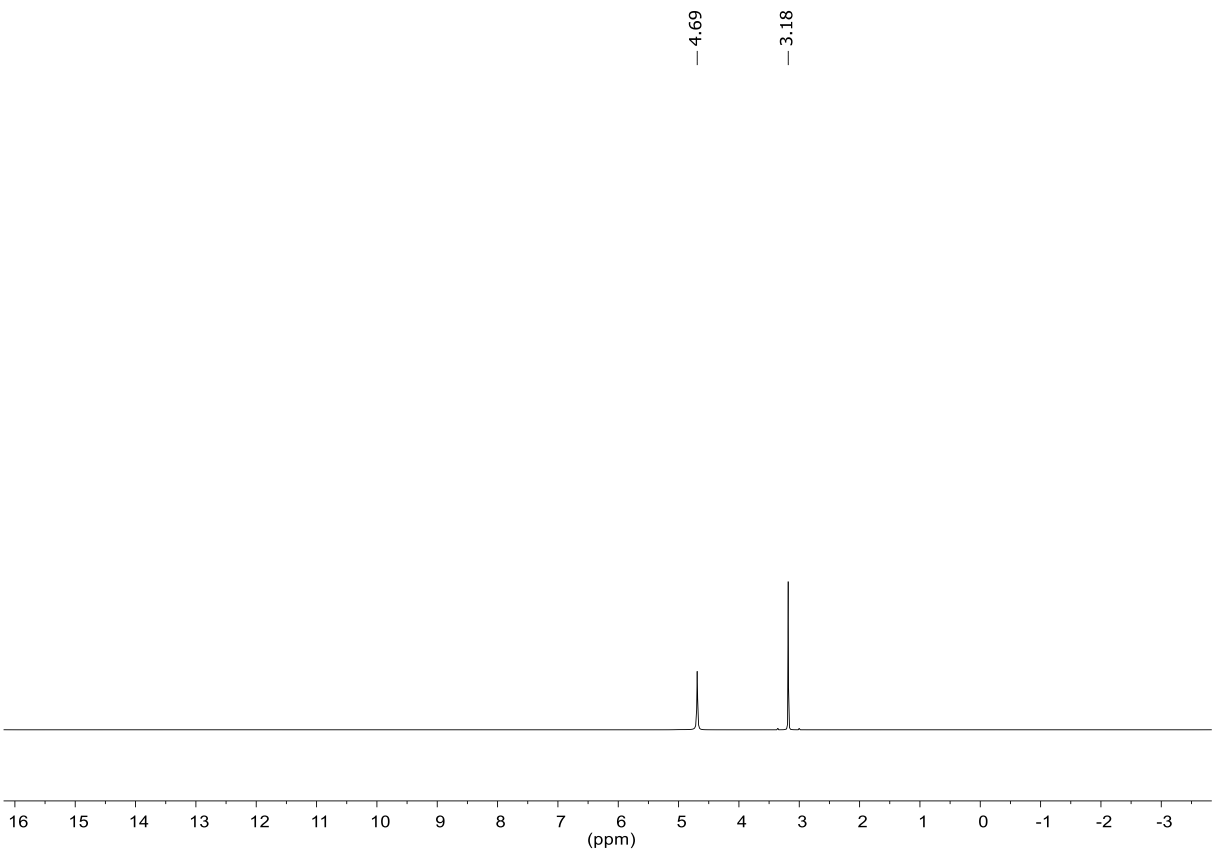

Figure S15. ^{1}H-NMR spectrum (400 MHz, water-d_2) of the polar methanol/water phase.

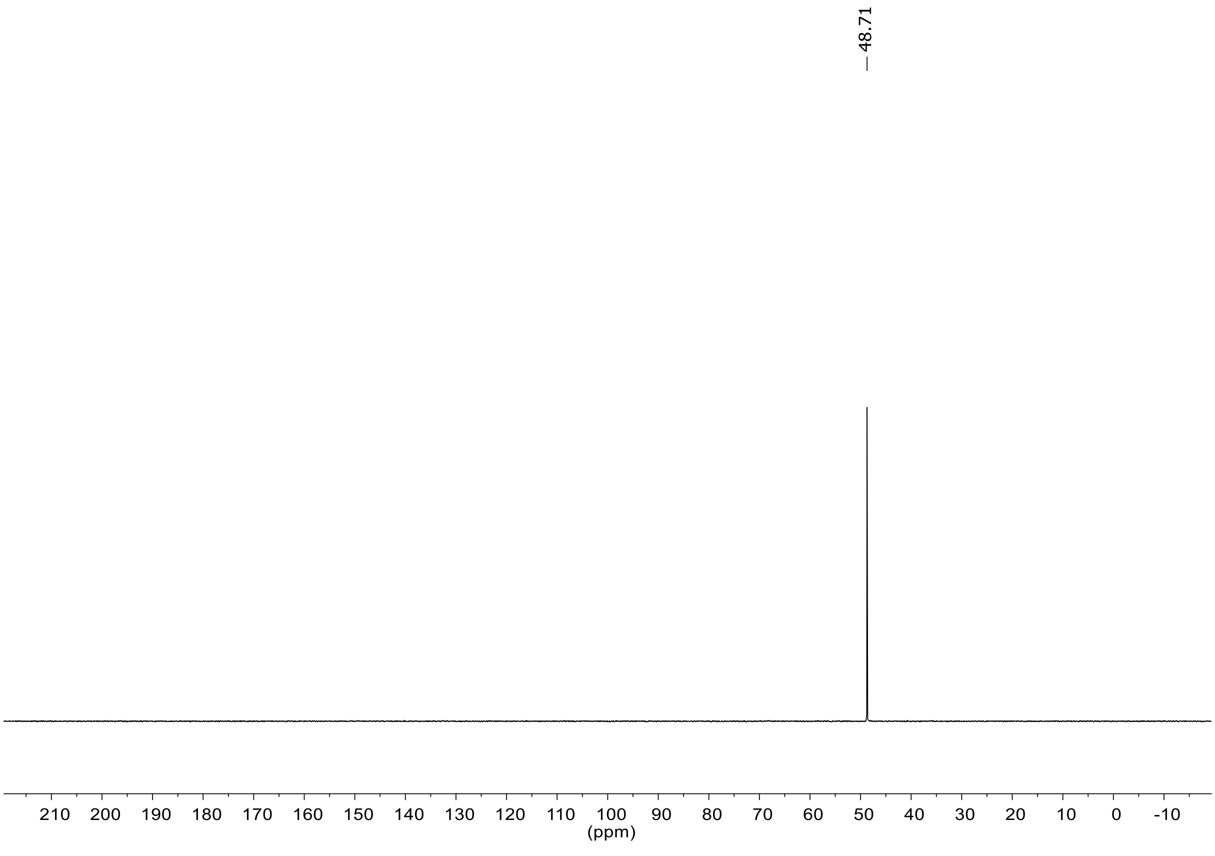

Figure S16. ^{13}C-NMR spectrum (101 MHz, water-d_2) of the polar methanol/water phase.

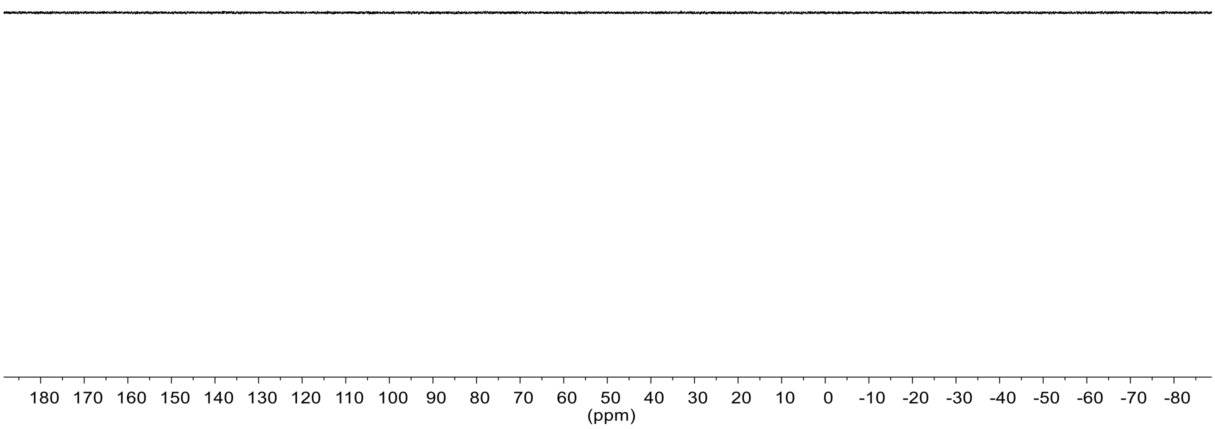

Figure S17. $^{31}P\{^{1}H\}$-NMR spectrum (162 MHz, water-d_2) of the polar methanol/water phase.

1.6 DMC hydrogenation experiments

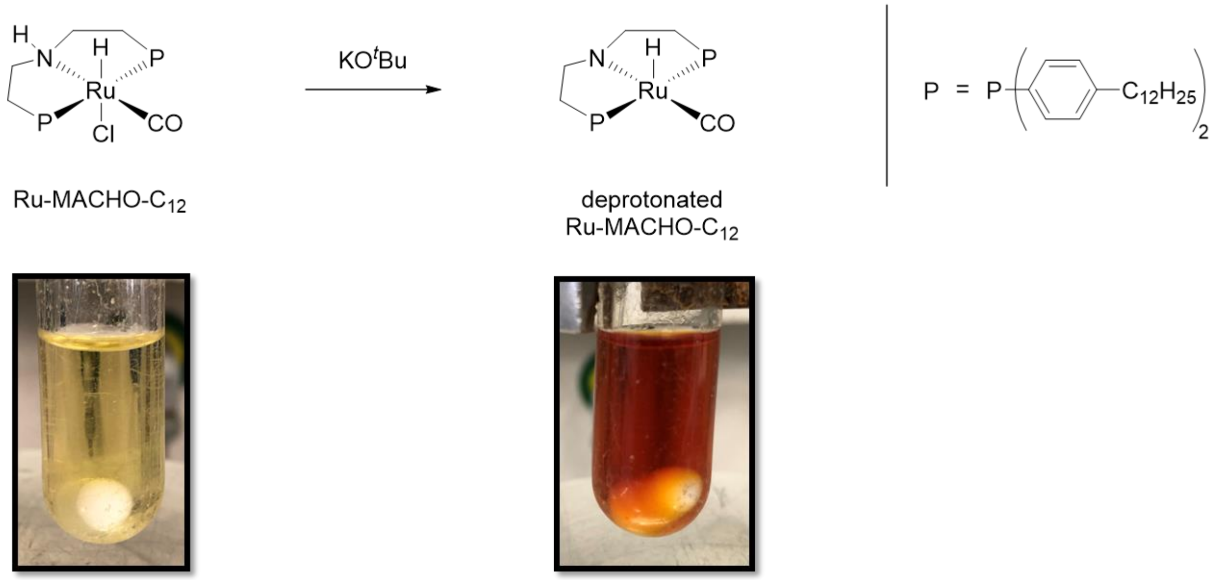

Figure S18. Activation of the Ru-precursor using KO^{t}Bu as base.

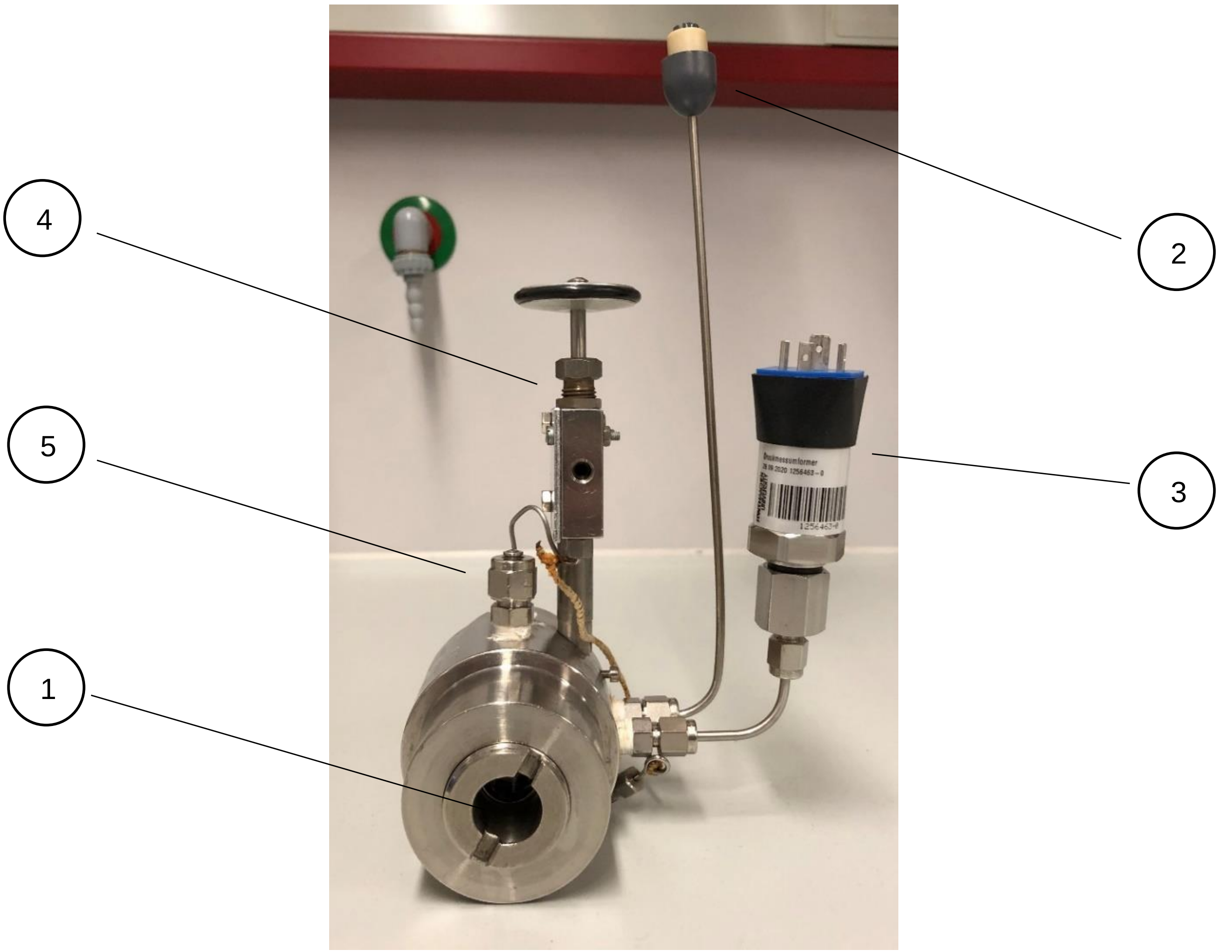

Figure S19. Picture of a 10 mL window reactor with sight glass (1), thermo element (2), digital pressure transducer (3), needle valve (4) and blind plug (5).

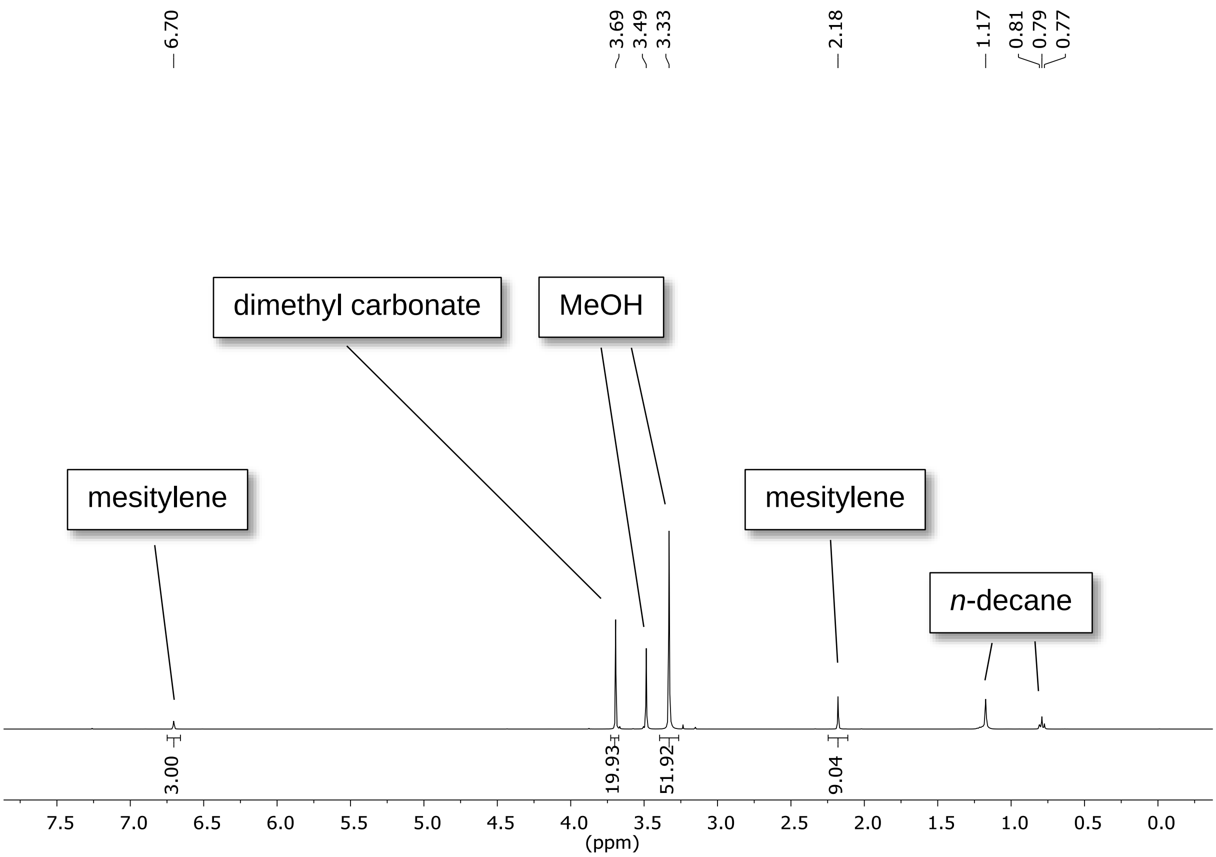

Figure S20: Typical ^{1}H-NMR spectrum (400 MHz, chloroform-*d*) of the product phase after DMC hydrogenation.

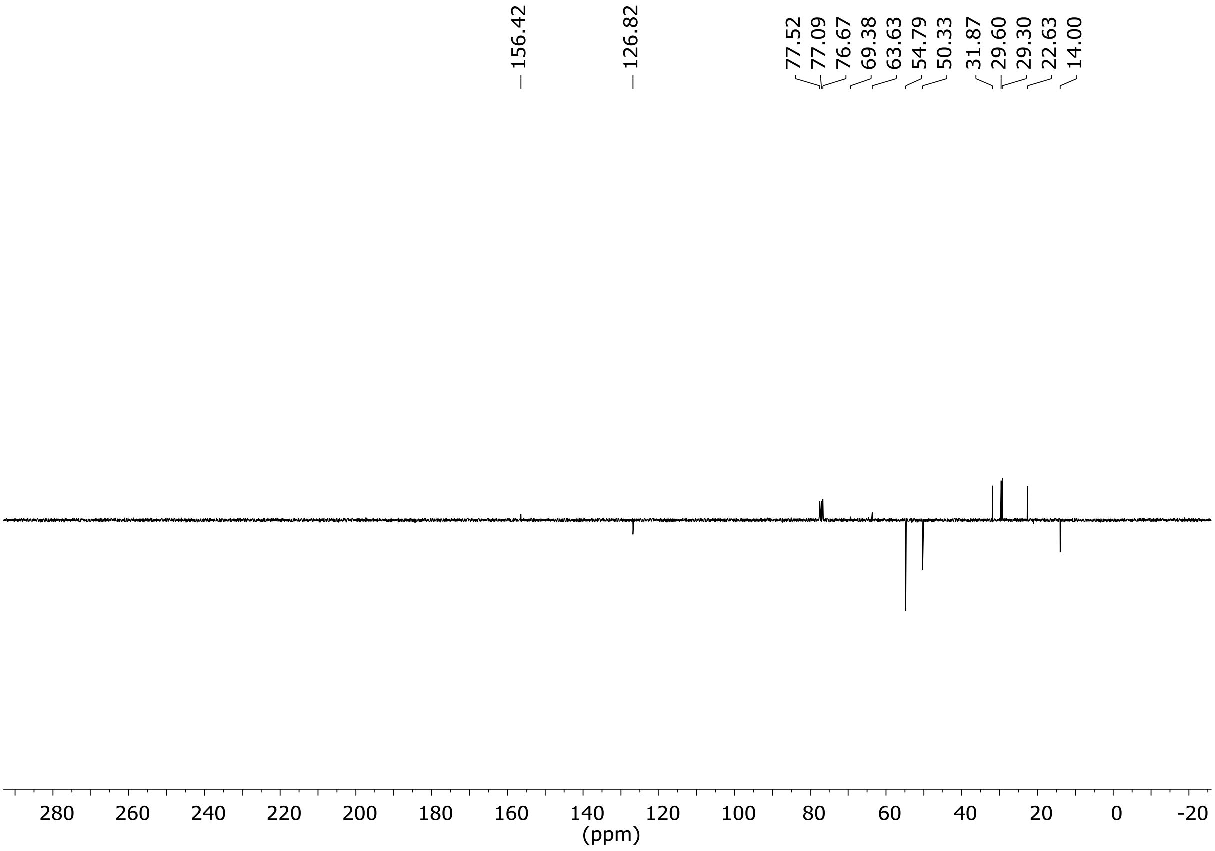

Figure S21. Typical ^{13}C-NMR spectrum (101 MHz, chloroform-*d*) of the product phase after DMC hydrogenation.

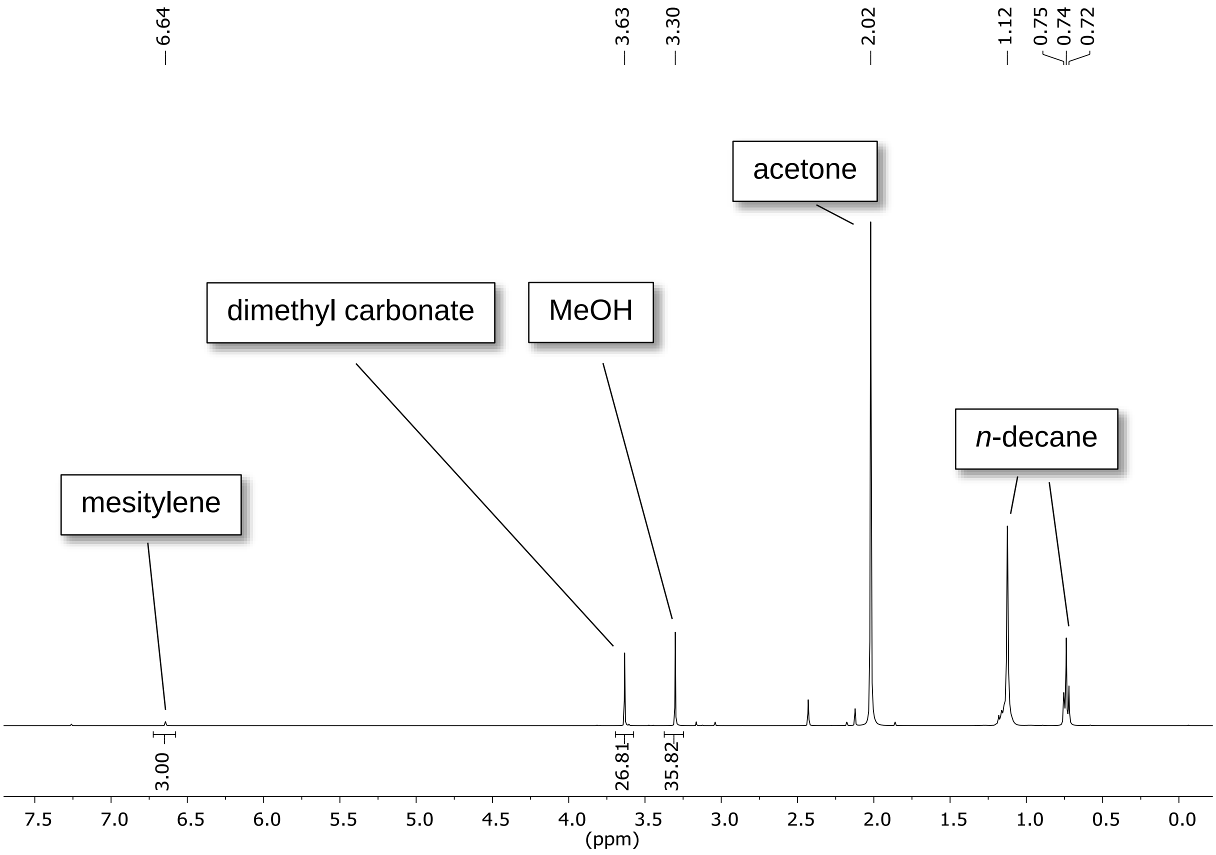

Figure S22. Typical ^{1}H-NMR spectrum (400 MHz, chloroform-*d*) of the catalyst phase after DMC hydrogenation.

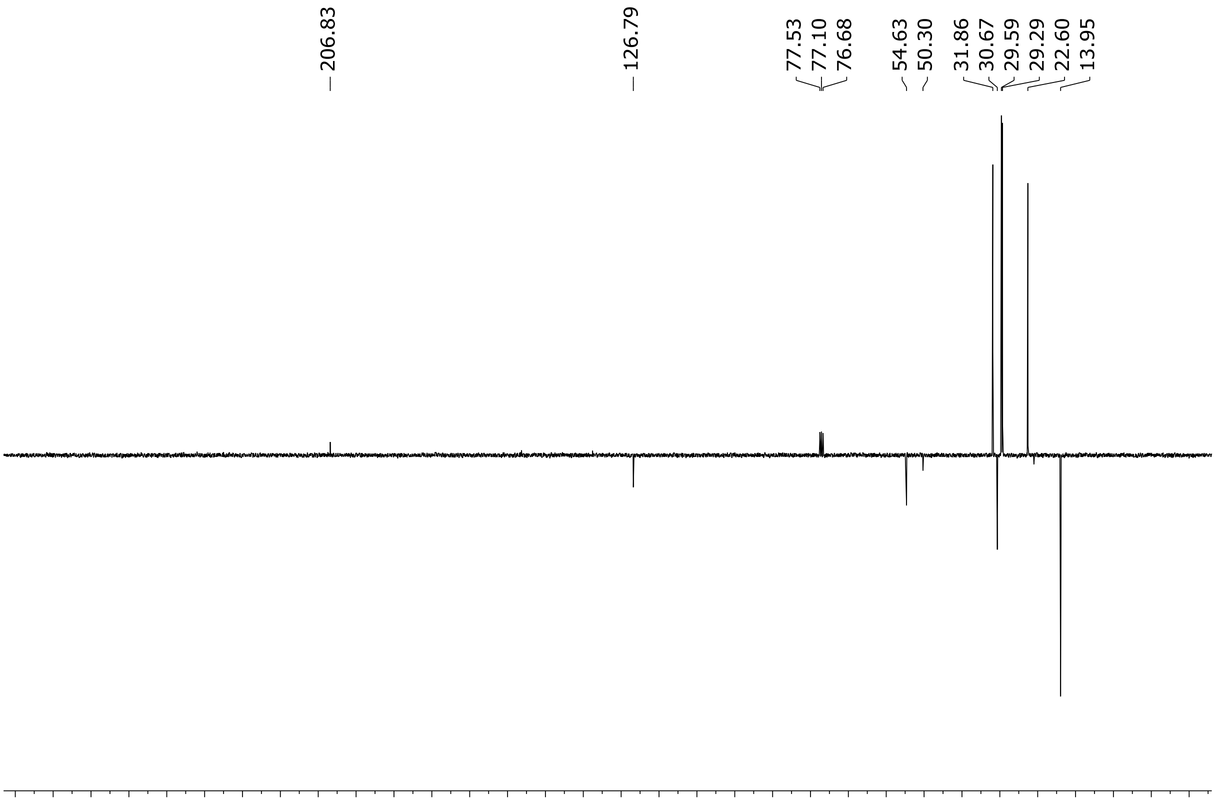

Figure S23. Typical ^{13}C-NMR spectrum (101 MHz, chloroform-*d*) of the catalyst phase after DMC hydrogenation.

1.7 CO_2 hydrogenation experiments

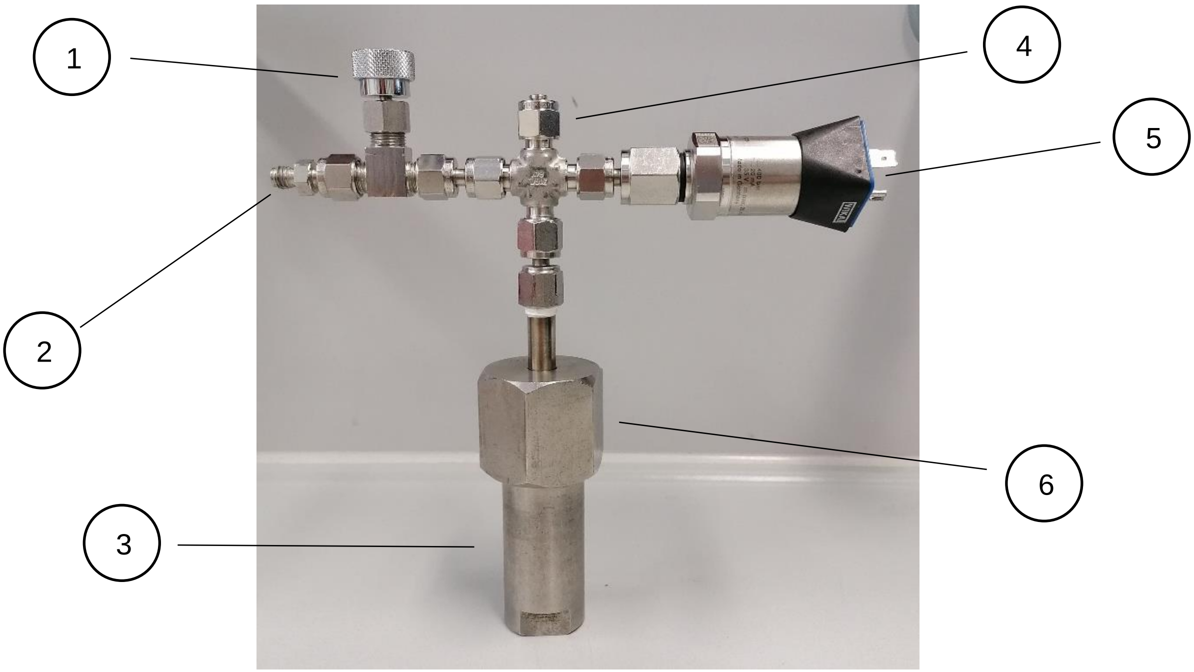

Figure S24. Picture of a 10 mL high pressure standard reactor without windows with needle valve (1), connection thread for gases (2), reactor pot (3), screw cap (for addition or removal of liquids) (4), digital pressure transducer and union nut (5).

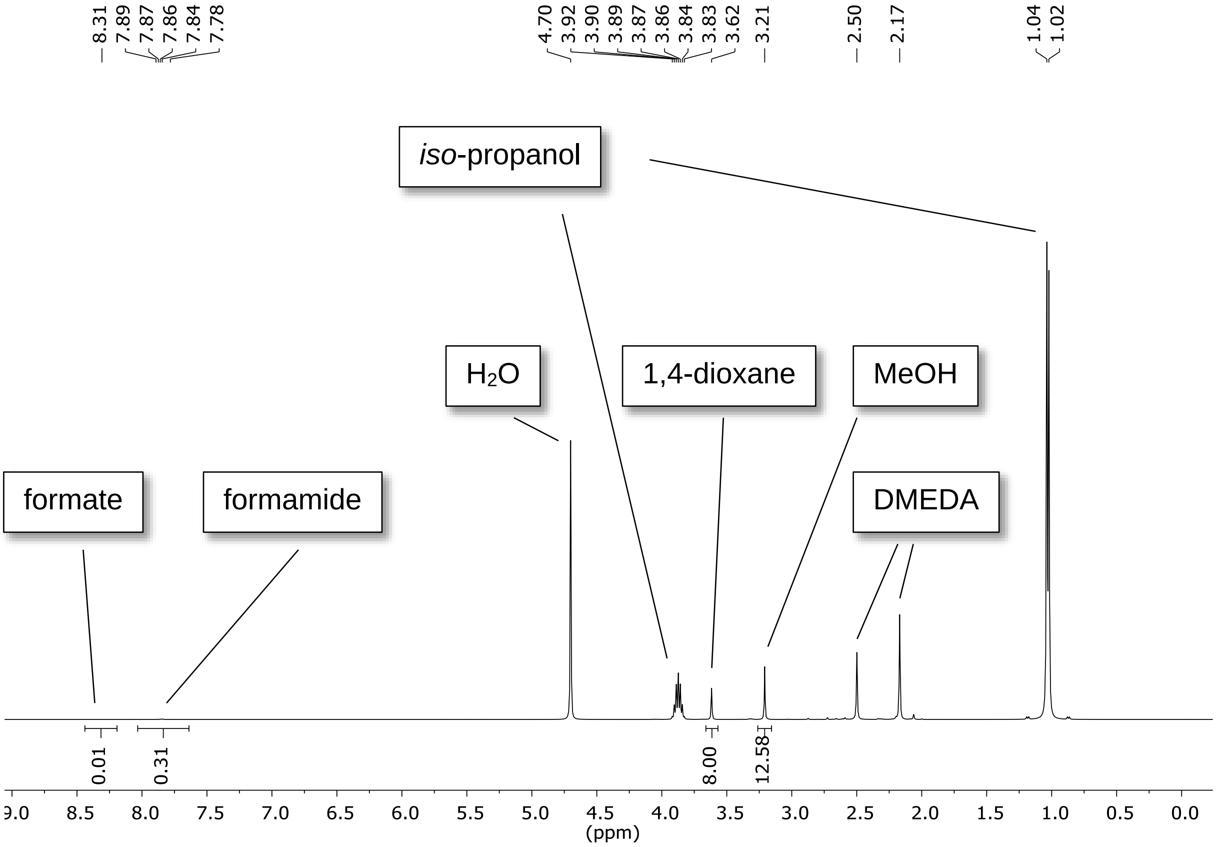

Figure S25. Typical ^{1}H-NMR spectrum (400 MHz, water-d_2) of the product phase after CO_2 hydrogenation.

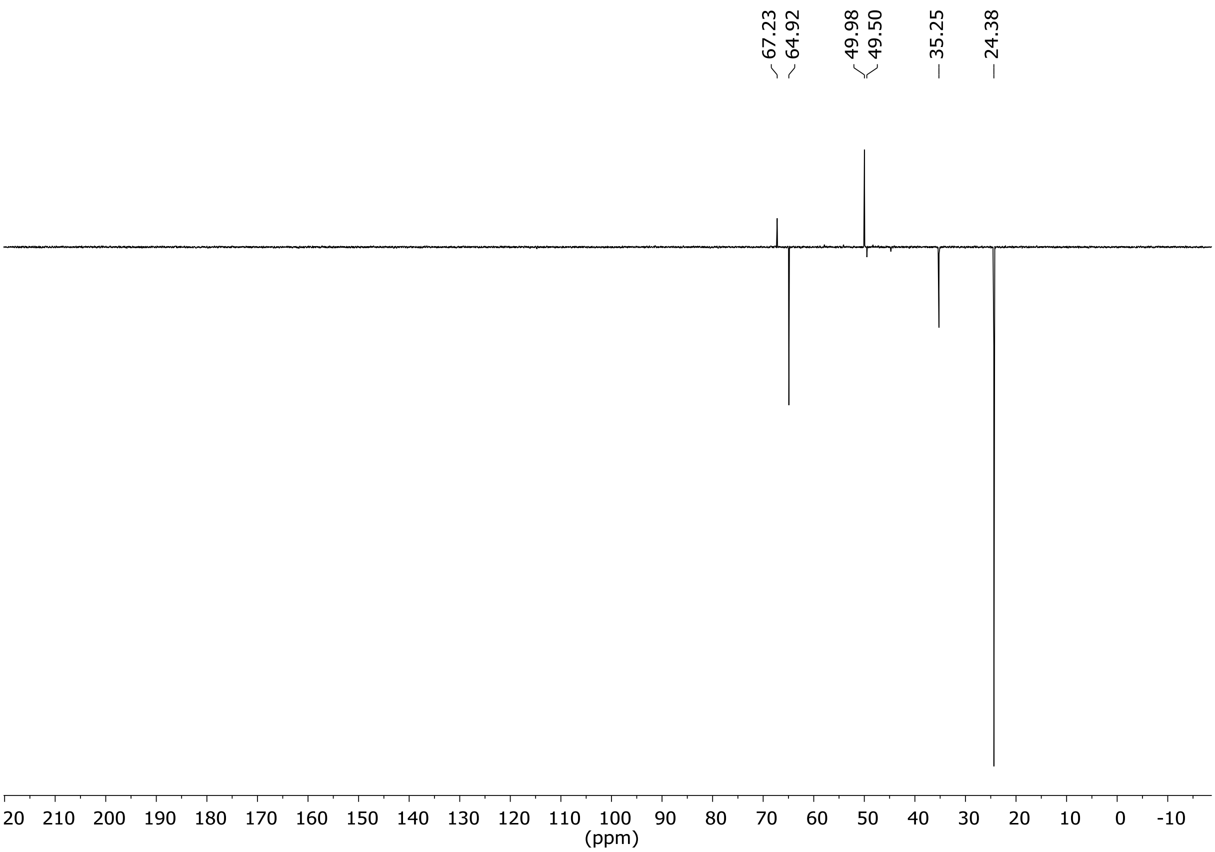

Figure S26. Typical ^{13}C-NMR spectrum (101 MHz, water-d_2) of the product phase after CO_2 hydrogenation.

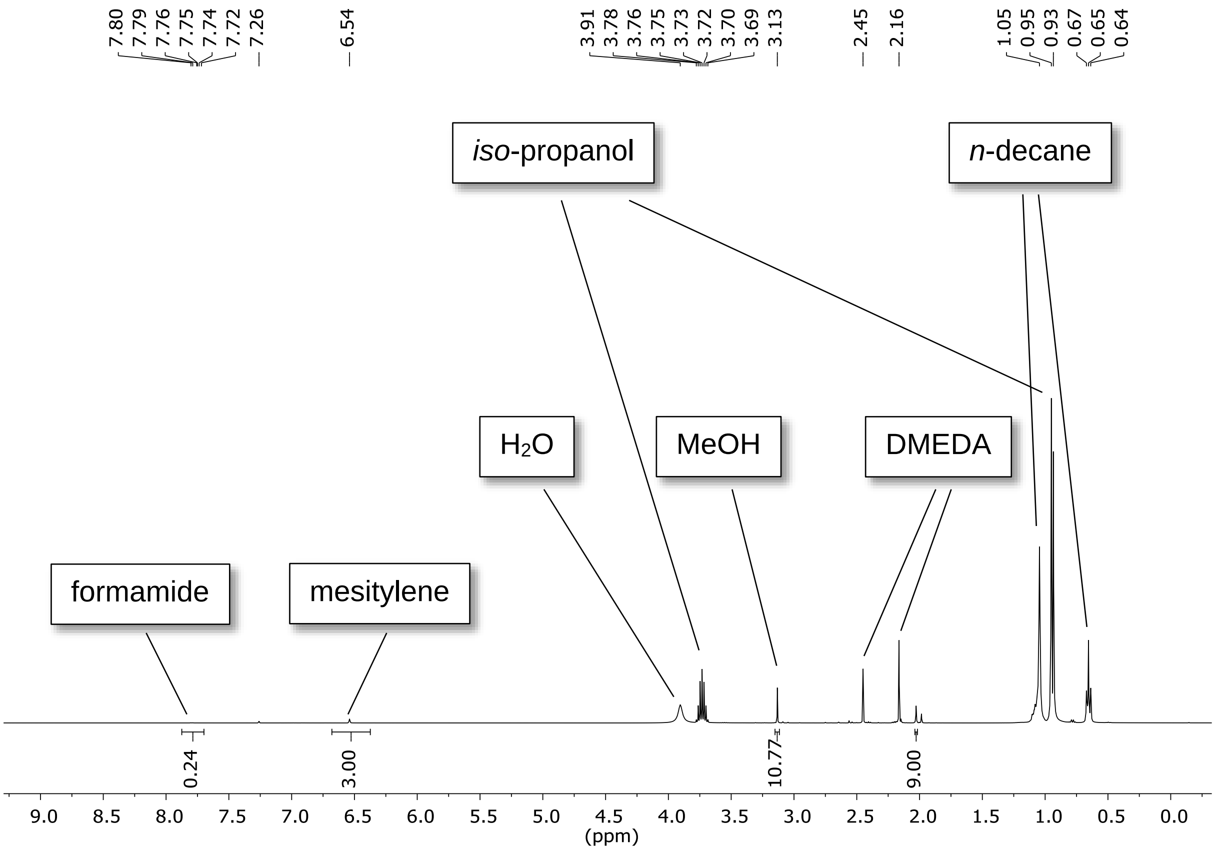

Figure S27. Typical ^{1}H-NMR spectrum (400 MHz, chloroform-*d*) of the catalyst phase after CO_2 hydrogenation.

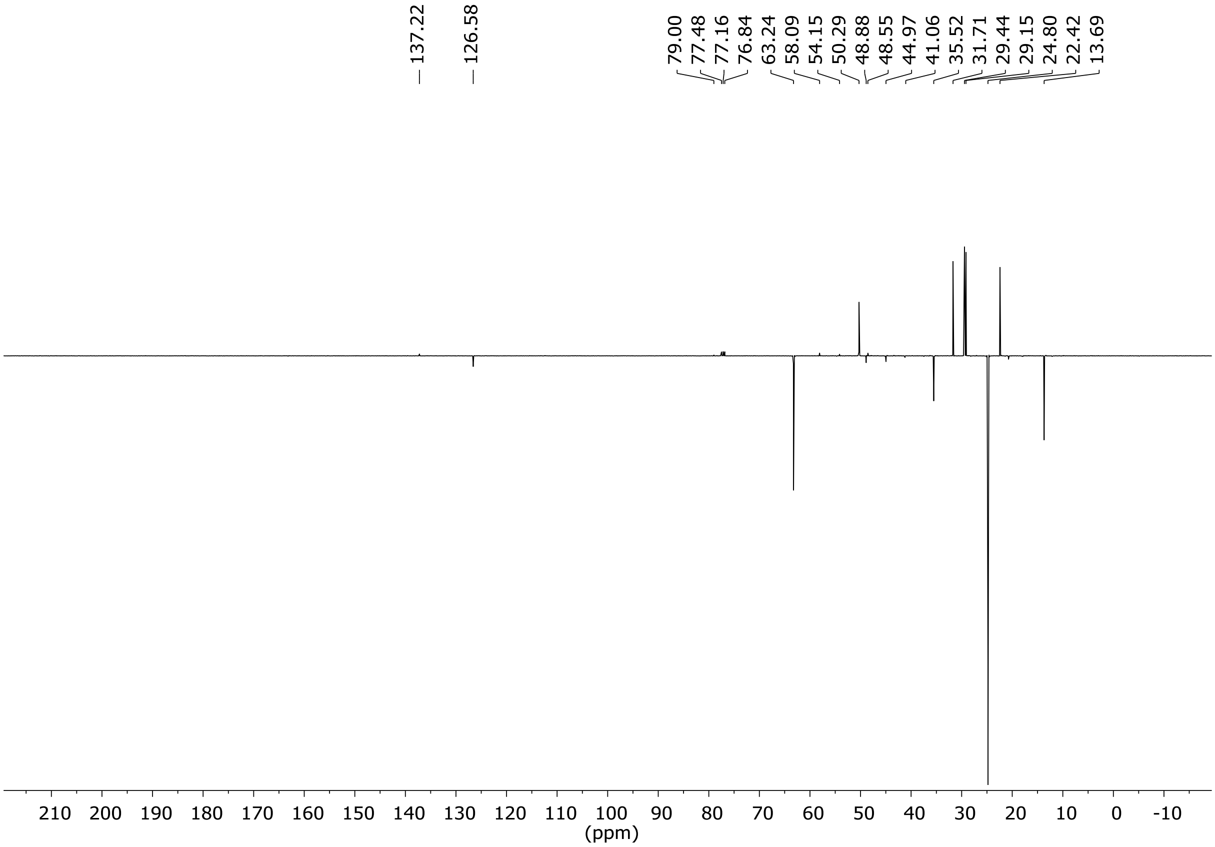

Figure S28. Typical ^{13}C-NMR spectrum (101 MHz, chloroform-*d*) of the catalyst phase after CO_2 hydrogenation.

1.8 Catalyst Recycling

Table S1. Recycling experiments for the liquid-liquid multiphasic hydrogenation of CO_2 to methanol via the amine-assisted pathway using the tailored Ru-MACHO-C_{12} catalyst in a window reactor (first series).[a]

Run	Ru leaching [%]	TON formamides	TON methanol
1	12.72	47	1200
2	7.67	123	1811
3	3.69	77	1901
4	1.77	72	1782
5	1.46	72	1736
6	1.09	68	1783
7	0.94	103	1706
8	0.83	73	2024
9	0.59	79	1669
10	0.58	56	1711
11	0.56	74	1912
	**31.90**[b]	**844**[b]	**19235**[b]

[a] Determined by ICP-MS measurements of the polar product phase and expressed as a percentage of the initial catalyst loading. [b] Total of all cycles.

Table S2. Recycling experiments for the liquid-liquid multiphasic hydrogenation of CO_2 to methanol via the amine-assisted pathway using the tailored Ru-MACHO-C_{12} catalyst in a window reactor (second series).[a]

Run	Ru leaching [%]	TON formamides	TON methanol
1	19.12	575	475
2	9.78	578	1110
3	3.64	410	1319
4	1.47	313	1425
5	0.83	165	1280
6	0.67	211	1592
7	0.50	141	1579
8	0.35	121	1703
9	0.27	92	1579
10	0.25	88	1644
11	0.26	114	2153
	**37.14**[b]	**2808**[b]	**15859**[b]

[a] Determined by ICP-MS measurements of the polar product phase and expressed as a percentage of the initial catalyst loading. [b] Total of all cycles.

Table S3. Recycling experiments for the liquid-liquid multiphasic hydrogenation of CO_2 to methanol via the amine-assisted pathway using the tailored Ru-MACHO-C_{12} catalyst in a standard high pressure reactor without windows (third series).[a]

Run	Ru leaching [%]	TON formamides	TON methanol
1	16.72	103	1230
2	3.80	177	1452
3	1.16	135	1371
4	0.56	119	1436
	**22.24**[b]	**534**[b]	**5489**[b]

[a] Determined by ICP-MS measurements of the polar product phase and expressed as a percentage of the initial catalyst loading. [b] Total of all cycles.
